# Iron allocation to chloroplast proteins depends on the DNA-binding protein WHIRLY1

**DOI:** 10.1007/s00425-025-04736-8

**Published:** 2025-06-17

**Authors:** Karin Krupinska, Susann Frank, Luca Boschian, Monireh Saeid Nia, Susanne Braun, Anke Schäfer, Ulrike Voigt, Ewa Niewiadomska, Bettina Hause, Götz Hensel, Wolfgang Bilger

**Affiliations:** 1 Institute of Botany, Christian-Albrechts-University (CAU), Kiel, Germany; 2 Heinrich Heine University Düsseldorf, Faculty of Mathematics and Natural Sciences, Centre for Plant Genome Engineering, and Cluster of Excellence in Plant Sciences, Heinrich Heine University Düsseldorf, Düsseldorf, Germany; 3 The Franciszek Górski Institute of Plant Physiology, Polish Academy of Sciences, Niezapominajek 21, 30-239 Krakow, Poland; 4 Department of Cell and Metabolic Biology, Leibniz Institute of Plant Biochemistry, Weinberg 3, 06120 Halle, Germany

**Keywords:** Ferritin, Iron cofactors, Iron proteins, *Hordeum vulgare* L., WHIRLY proteins

## Abstract

**Main conclusion:**

The DNA-binding protein WHIRLY1, sharing structural similarities with ferritin, plays a role in the formation of iron cofactor proteins within chloroplasts.

**Abstract:**

Previous studies indicated that barley plants with a knockdown of *HvWHIRLY1* containing a minimal amount of the protein are compromised in chloroplast development and photosynthesis, and get chlorotic leaves when grown at high irradiance. Thereby, the leaves display signs of iron deficiency. Metal determination revealed, however, that leaves of WHIRLY1-deficient plants had a regular iron content. Nevertheless, WHIRLY1-deficiency affected the functionality of photosystem II less than that of photosystem I, which has a higher demand for iron. Immunological analyses revealed that components of both photosystems had reduced levels. Additionally, the levels of other chloroplast proteins containing different classes of iron cofactors were lower in the WHIRLY1-deficient plants compared to the wild type. In contrast, the level of the iron sequestering protein ferritin increased in WHIRLY1-deficient lines, whereby high irradiance intensified this effect. RNA analyses showed that the upregulation of ferritin coincided with an enhanced expression of the corresponding gene, reflecting an apparent overload of chloroplasts with free iron. Ferritin and WHIRLY proteins are known to share the same oligomeric structure. Therefore, the high abundance of ferritin in WHIRLY1-deficient plants might be a compensation for the reduced abundance of WHIRLY1. Enhanced expression levels of genes encoding photosynthesis proteins and iron cofactor proteins indicate a demand for protein formation or assembly of protein complexes. The results support a general role of WHIRLY1 in assembly and/or stabilization of chloroplast proteins and, moreover, suggest a specific function in sequestering and supply of iron in chloroplasts.

**Supplementary Information:**

The online version contains supplementary material available at 10.1007/s00425-025-04736-8.

## Introduction

WHIRLY proteins are multifunctional DNA-binding proteins found in all DNA-containing plant cell compartments (Krupinska et al. 2022). They affect multiple developmental processes and stress resistance of plants (Krupinska et al. 2022). Most plants contain two WHIRLY proteins, whereby WHIRLY1 is targeted to chloroplasts and also appears in the nucleus after relocation from chloroplasts (Grabowski et al. 2008; Isemer et al. 2012). In contrast to most plant species, *Arabidopsis thaliana* has three WHIRLY proteins whereby WHIRLY1 and 3 are targeted to chloroplasts. *WHIRLY1/3* mutants of Arabidopsis showed variegated leaves (Maréchal et al. 2009), which were observed more frequently when plants were grown in high light (Guan et al. 2018). Such variegation might be caused by unequal resistance of individual plastids to photooxidative stress, as reported for the *immutans* mutant of Arabidopsis (Yu et al. 2007). In maize, transposon-induced mutations of *WHIRLY1* led to seedlings with ivory leaves that did not survive after reaching the four-leaf stage (Prikryl et al. 2008). Likewise, *WHIRLY1* mutants of rice produced by CRISPR Cas9-mediated genome editing cannot survive after the seedling stage (Qiu et al. 2022), indicating that WHIRLY1 is essential for chloroplast development. *WHIRLY1* knockdown plants of barley with a reduced abundance of chloroplast-nucleus located WHIRLY1 showed delayed chloroplast development (Krupinska et al. 2019). Chloroplast development was even more delayed in barley knockout mutants prepared by CRISPR Cas9-mediated knockout of *WHIRLY1* (Krupinska et al. 2024). Although the *HvWHIRLY1* knockdown plants had regular chlorophyll content during growth at low irradiance, they got chlorotic when grown at high irradiance (Swida-Barteczka et al. 2018; Saeid Nia et al. 2022). Electron spin resonance measurements revealed that the bleached leaves accumulated higher levels of reactive oxygen species (ROS) than wild-type leaves (Swida-Barteczka et al. 2018). The irradiance-dependent bleaching of the leaves coincided with a decline in photosystem II (PSII) efficiency (F_V_/F_M_) and an increase in the pool of the xanthophyll cycle pigments violaxanthin, antheraxanthin, and zeaxanthin (VAZ) as well as in the de-epoxidation state (DEPS) (zeaxanthin and antheraxanthin are formed at the expense of violaxanthin) of the VAZ pool (Swida-Barteczka et al. 2018). These features are also typical for chlorosis induced by iron deficiency (Morales et al. 1990) resulting from activating an iron economy program involving changes in photosynthesis, protein levels, and gene expression (Hantzis et al. 2018). Consistent with the light-dependent increase in VAZ pool pigments and in DEPS, the increase in non-photochemical quenching under iron-deficient conditions (Morales et al. 1990; Saito et al. 2010) serves in photoprotection under these circumstances (Morales et al. 1994).

The impact of iron deficiency on photosynthesis and its serious consequences for agricultural production have been studied for a long time (Terry and Zayed 1995). The reason underlying the negative consequences of iron deficiency for photosynthesis is due to its high demand for iron cofactors. Indeed, chloroplasts are the major sinks for iron in plants and most of the iron is found in components of electron transport, such as the iron–sulfur cluster containing proteins of photosystem I (PSI), ferredoxin, the Rieske protein, and the iron-containing heme groups of the cytochrome b_6_f complex and of cytochrome b_559_ in PSII (Briat et al. 2015; Hantzis et al. 2018). Chlorosis induced by iron deficiency furthermore affects nitrogen and sulfur metabolism which depend on iron–sulfur cluster cofactors being produced in chloroplasts by the sulfur utilization factor (SUF) machinery (Kroh and Pilon 2020). In consequence, nitrogen content and dry weight of leaves decrease, leading to a reduction in biomass and quality of plants (Briat et al. 2015).

Since the chlorotic phenotype of WHIRLY1-deficient plants resembles that of iron-deficient plants, this study addressed the question whether WHIRLY1-deficient plants indeed suffer from iron deficiency. Surprisingly, the leaves of barley plants with only minimal amounts of WHIRLY1 had regular iron levels. Nevertheless, they displayed typical signs of iron deficiency that were intensified by high irradiance. The reduced levels of proteins containing different forms of iron cofactors suggest that WHIRLY1 is involved in the allocation of iron for the assembly of different iron cofactor proteins, thereby promoting chloroplast development and photosynthesis.

## Materials and methods

### Plant material and growth conditions

Barley grains (*Hordeum vulgare* L., cv. Golden Promise) were sown on soil (Einheitserde ED73, Einheitswerk Werner Tantau, Ütersen, Germany) and transferred for 3 days in a dark and cold chamber (6 °C) to synchronize germination. The imbibed grains were transferred to climate chambers where they were exposed either to 16 h light (150 μmol m^−2^ s^−1^, 21 °C) and 8 h darkness (18 °C) or to continuous light of different irradiances (50, 120, 200, or 350 μmol m^−2^ s^−1^) (Swida-Barteczka et al. 2018). Primary foliage leaves of the seedlings were analyzed at 10 days after sowing (das).

### Determination of P_M_ and F_V_/F_M_

P_700_ (P_M_) and F_V_/F_M_ were measured using a scripted program with a Dual-PAM-100 measuring system (Heinz Walz GmbH, Effeltrich, Germany). Briefly, the leaves were placed in the instrument, so that the measuring light irradiated the central portion of the leaf. To avoid artifacts due to different leaf dimensions, a frame was made from black cardboard, that limited the measuring window to a specific size (4 × 17 mm). 30 s after the determination of F_V_/F_M_ and following 15 s pre-irradiation with far-red light, P_M_ was determined. The plants were dark adapted for 1 h, followed by exposure to at least 20 min room light (5 µmol m^−2^ s^−1^).

### Isolation of thylakoid membranes and separation of protein complexes

Chloroplasts were isolated from primary foliage leaves of barley grown for 7 days in a light (50 µmol m ^2^ s^−1^ or 150 m ^2^ s^−1^)/dark cycle of 18/6 h. Thylakoid membranes were isolated as described (Mullet et al. 1980). Membranes (1 mg of chlorophyll per ml) were solubilized for 10 min with 1% (w/v) dodecyl-ß-D-maltoside (Sigma-Aldrich, St. Louis, MO, USA) on ice. After 5 min of centrifugation at 20,000 g, aliquots were applied to 0.4–0.85 M sucrose gradients prepared as described by Jensen et al. (2000). Gradients were centrifuged for 24 h at 200,000 g. Thereafter, gradients were fractionated, and the fractions were used for pigment determination and immunoblot analysis.

### Immunoblot analyses

SDS-PAGE was either performed with leaf extracts or aliquots of sucrose gradient fractions. Leaves were ground in liquid nitrogen. Total proteins were extracted from leaf powder as reported (Krupinska et al. 2014). Equal protein amounts were subjected to SDS-PAGE on 10–16% (w/v) polyacrylamide gels. Proteins were transferred onto nitrocellulose (HP40.1, Roth, Karlsruhe, Germany) by semi-dry electroblotting (Humbeck et al. 1996). Antibodies against WHIRLY1 (AS163953), PsaC (AS10 939), PsbA/D1 (AS01016), PsbD/D2 (AS06146), LHCB1 (AS01004), LHCB5 (AS01009), LHCA1 (AS01005), RbcL (AS03037), plastocyanin (AS06141), aconitase (AS09521), ferredoxin (AS06121), ferritin (AS152898), catalase (AS09501), iron superoxide dismutase FeSOD (AS06125), and copper-zinc superoxide dismutase Cu/ZnSOD (AS06170) were purchased from Agrisera (Vännäs, Sweden). An antibody specific for cytochrome b559 was prepared by O. Vallon (Vallon et al. 1987). An antibody directed toward the complete photosystem I core complex was provided by P.E. Jensen (Copenhagen University, Denmark). LHCII was detected by an antibody provided by A. Melis (Harrison and Melis 1992). The antibody directed toward cytochrome f (PetA) was a gift from Richard Malkin (Bruce and Malkin 1991). The antibody toward nitrite reductase (ab243179) was purchased from Abcam (Amsterdam, The Netherlands) and the antibody toward hydroxymethylenyl disphosphate synthase abbreviated HDS (PHY0887S), purchased from PhytoAB (San Jose, CA, USA). The antibody against lipoxygenase 2 (LOX2) was used as described in Bachmann et al. (2002). Immunoreactive complexes were visualized using a peroxidase coupled secondary antiserum with chemiluminescence detection (ECL Select, Amersham, Pierce Thermo Scientific, Waltham, MA, USA). The immunoblots were imaged using a film (Amersham Hyperfilm ECL, GE Healthcare, Freiburg, Germany) or the ChemiDoc system (BioRad, Hercules, CA, USA).

### Determination of mRNA levels by quantitative RT-PCR

RNA was extracted from the same leaf powder used for protein analyses employing the peqGOLD-TriFast reagent (Peqlab Biotechnology, Erlangen, Germany) and was used for the synthesis of cDNA with the Quanti Tect^®^ Reverse Transcriptase Kit (Qiagen, Hilden, Germany) according to the manufacturer’s protocol. qRT-PCR was done with gene specific primers (Supplementary Table S1). Data analysis was accomplished by the Rotor-Gene Q software (version 2.0.2.4) (Qiagen). Relative quantification of transcript levels was performed as described before (Krupinska et al. 2019). Both, plastid transcripts and nuclear transcript data, were normalized to the level of the GAPDH mRNA (Rapacz et al. 2012).

### Determination of metals

Concentrations of iron, zinc, copper, and manganese in leaves were quantified using inductively coupled plasma-mass spectroscopy (ICP-MS) with an Agilent Technologies 7700 instrument (Böblingen, Germany) (Jezek et al. 2015).

## Results

### Characterization of seedlings grown under standard conditions

#### Functionality and abundance of photosystem I are reduced in WHIRLY1-deficient plants

In the previous studies, WHIRLY1-deficient barley plants obtained by RNAi-mediated knockdown of *HvWHIRLY1* were shown to be impaired in growth, chloroplast development, photosynthesis, light acclimation, and oxidative stress resistance (Swida-Barteczka et al. 2018; Krupinska et al. 2019; Saeid Nia et al. 2022, 2023). To further investigate the reasons underlying the plants’ reduced capabilities, the functionality and composition of the photosynthetic apparatus were investigated under different growth conditions. Initially, the plants were grown in a daily light/dark cycle that was supposed to impose no stress on the plants.

**Fig. 1:**
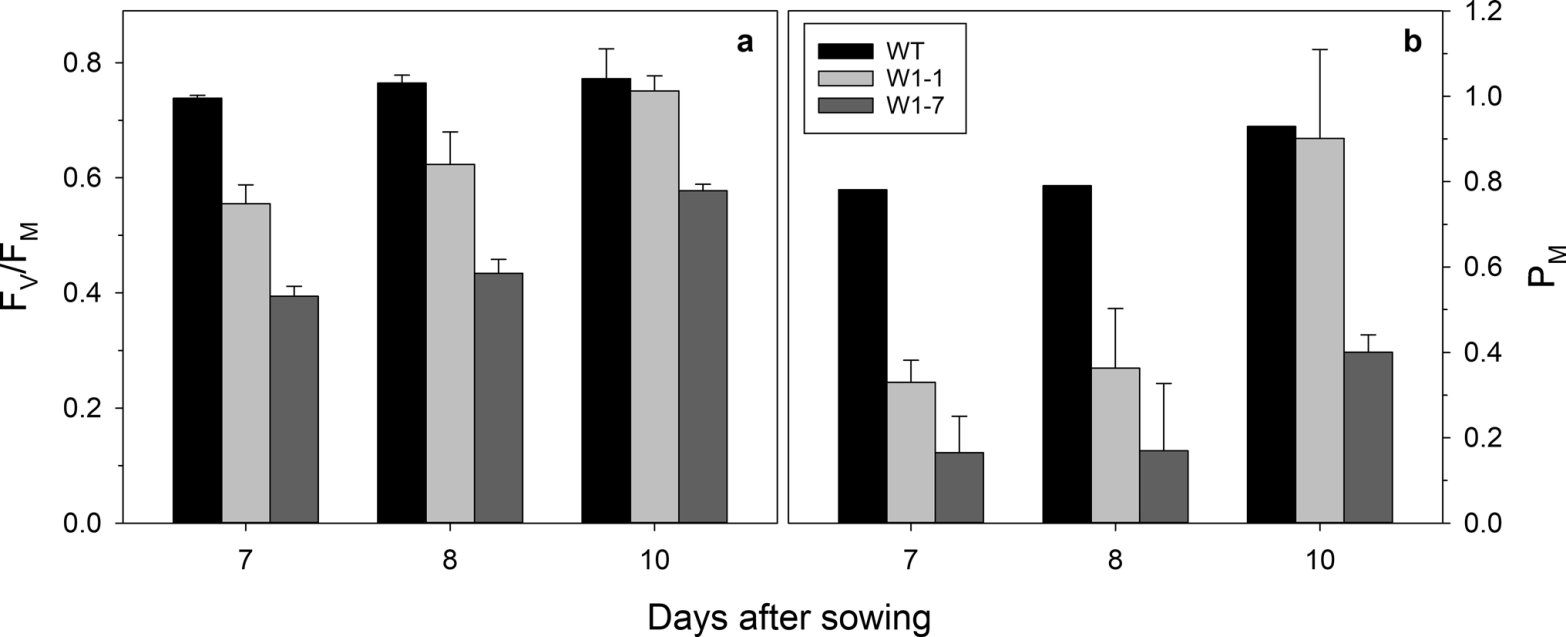
Efficiencies of the photosystems. **a** The maximal quantum yield of PS II, F_V_/F_M_ and **b** The maximal absorbance change of the photosystem I reaction center P700 (P_M_) were measured with primary foliage leaves of the wild type (WT), the W1-1 and the W1-7 lines grown under standard conditions. Depicted values are means ± standard deviation of *n* = 9–11 leaves from three independent experiments, each comprising 3–4 leaves

As reported previously, the two barley lines with a knockdown of *WHIRLY1* (W1) accumulated only 1% (W1-7) or 10% (W1-1) of the wild-type WHIRLY1 level (Krupinska et al. 2014). Despite a distinct difference in F_V_/F_M_ between wild type and the RNAi-W1 plants at early stages of leaf development, the F_V_/F_M_ values of W1-1 and wild-type (WT) leaves were almost identical at 10 days after sowing (das) (Fig. 1a). In leaves of the W1-7 seedlings, the development associated increase in the efficiency of PSII, represented by F_V_/F_M_ values, needed more time than in the WT and the W1-1 line. Despite this difference, the data follow a previous report characterizing the WHIRLY1-deficient barley plants (Krupinska et al. 2019). Both studies indicated that the abundance of WHIRLY1 determines the build-up of PSII functionality.

In addition, in this study, leaves of the three genotypes were characterized by the PSI reaction center functionality, as measured by light-induced oxidation of P_700_. The amount of oxidizable P_700,_ as indicated by the maximal absorbance change at 820 nm (P_M_), increased during development in all three genotypes, particularly from 7 until 10 das (Fig. 1b). While in leaves collected at 10 das, the amounts of P_700_ of the wild-type leaves and those of the W1-1 line were close to each other, the P_700_ content of the W1-7 line was reduced by more than 50% (Fig. 1b). The W1-7 line and the wild type were furthermore used for measurements of the time-dependent changes in the redox state of P_700_, plastocyanin (PC) and ferredoxin (FD) (Supplementary Fig. S1). In line with the reduced P_M_ values, the amplitudes of the redox changes of the three components were lower in WHIRLY1-deficient plants compared to the wild type. 77 K fluorescence emission spectra revealed that the long-wavelength fluorescence of PSI was shifted to shorter wavelengths (from 735 to 732–733 nm) (Supplementary Fig. S2). Such a shift in the long-wavelength fluorescence emission, which can be assigned to the light-harvesting complex of PSI (LHCA) (Jensen et al. 2007), indicates a problem either in the assembly or the stability of PSI (Landau et al. 2009).

#### Separation of thylakoid membrane complexes by sucrose density gradient centrifugation

**Fig. 2:**
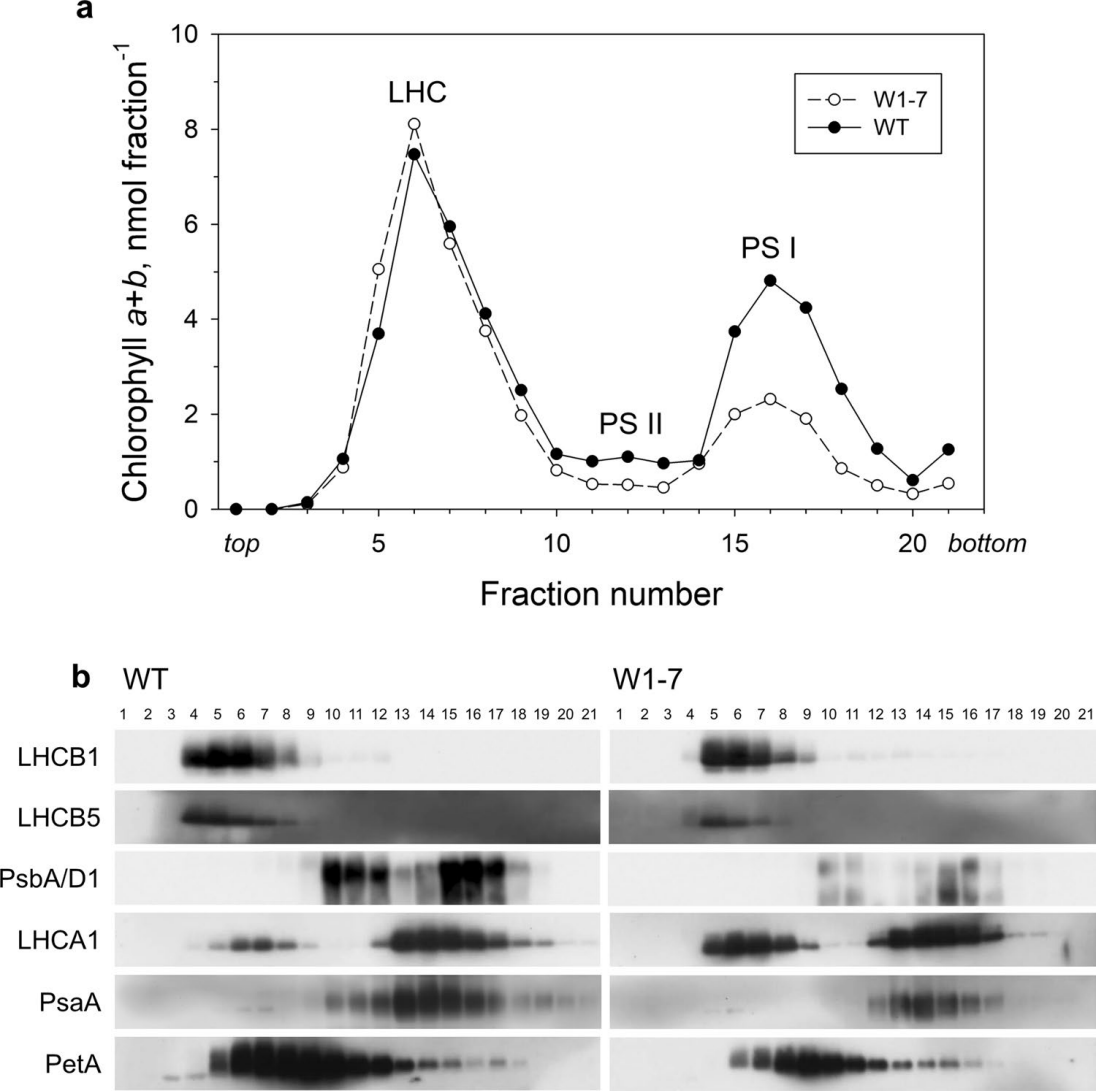
Separation of thylakoid membrane complexes by sucrose density gradient centrifugation. Thylakoids prepared from the wild type (WT) and *WHIRLY1* knockdown plants (W1-7), respectively, were solubilized with ß-maltoside and the resulting complexes were separated by sucrose density gradient centrifugation. **a** The distribution of chlorophyll was analyzed by HPLC analyses of the pigments extracted from the individual 21 fractions whereby fraction 1 is the top fraction. **b** Immunological analyses of photosynthesis-associated proteins (LHCB1, LHCB5, PsbA/D1, PsaA, LHCA1, and PetA) in the same fractions

The functional analyses with primary foliage leaves revealed that PSI was more affected by WHIRLY1 deficiency than PSII. To investigate the relative abundance and stability of individual complexes of the photosynthetic apparatus, thylakoid membranes prepared from wild-type and W1-7 chloroplasts were solubilized using beta-dodecyl maltoside, and the membrane complexes were subsequently separated by sucrose density gradient centrifugation as described by Jensen and co-workers (Jensen et al. 2000; Powikrowska et al. 2014). Thylakoid membrane samples from the two genotypes, respectively, were adjusted to identical amounts of chlorophyll before they were put onto the sucrose gradients yielding three green bands after centrifugation. The gradients were each separated into 21 fractions of equal volume for immunological analyses. The chlorophyll content of each fraction was determined in acetone extracts. As reported before, most of the chlorophyll was contained in the first band from the top of the gradient primarily containing light-harvesting complexes (LHC) (Powikrowska et al. 2014). The green band with the highest density is known to contain PSI (Jensen et al. 2000), and the band with the lowest chlorophyll content migrating between LHC and PSI represents PSII (Powikrowska et al. 2014). A comparison of the chlorophyll distribution in the different gradients revealed that the proportion of chlorophyll contained in the upper band (fractions 3–8) was relatively higher in the samples of the W1-7 line compared to the samples of the wild type, while the relative chlorophyll content of the bands containing PSI (fractions 14–18) and PSII (fractions 11–13) was reduced in the gradient obtained from W1-7 thylakoids (Fig. 2a). The differences in the relative abundances of the photosystems and the LHC were also visible in immunoblots performed with identical aliquots of the fractions and prepared simultaneously with the samples from the wild-type gradient and the W1-7 gradient (Fig. 2b). Based on the exact protein amounts, the levels of the reaction centers of PSII (PsbA/D1) and PSI (PsaA) as well as of cytochrome f (PetA) and of LHCB5 were reduced in the W1-7 line compared to the wild type (Fig. 2b). For comparison, the level of the major LHC associated with PS II (LHCB1) was rather stable.

In Arabidopsis, it has been found that WHIRLY1 interacts with LHCA1, which is a subunit of the light-harvesting complex attached to PSI (LHCA) (Huang et al. 2017). To compare the relative abundance of the photosystem I core complex and the LHCA antenna, fractions from the gradients were immunologically analyzed with an antibody specific to LHCA1. While the abundance of the core complex was reduced, the abundance of LHCA1 was apparently enhanced in thylakoids from the WHIRLY1-deficient plants. Moreover, the immunoblots showed that in the WHIRLY1-deficient plants the upper LHC containing band of the W1-7 gradient (fractions 3–8) contained an enhanced portion of LHCA1 compared to the wild-type gradient. This further indicates that LHCA coupling to the core complex of PSI is reduced in the W1-7 plants, being in line with the 77 K fluorescence emission spectra (Supplementary Fig. S2). When alternatively clear native gel electrophoresis (Järvi et al. 2011) was used for the separation of thylakoid membrane complexes, in W1 plants part of the LHCA1 appeared in a fraction/band of high motility, while in the wild type, all LHCA1 was contained in a fraction of lower motility also containing PSI core components (Supplementary Fig. S3). This result aligns with the finding that LHCA1 appeared as disconnected from PSI in the upper fraction of the sucrose gradient obtained with thylakoid membrane complexes from W1-7 chloroplasts compared to wild-type chloroplasts (Fig. 2).

#### The response of the photosynthetic apparatus to growth in continuous light of different irradiances

As described in a previous study, the build-up of the photosynthetic apparatus is delayed in WHIRLY1-deficient plants (Krupinska et al. 2019). The analysis here performed with plants grown under the same standard conditions revealed furthermore that during leaf development and even in mature leaves, WHIRLY1-deficient plants showed a reduced maximal redox change of PSI. At the same time, PSII efficiency as measured by F_V_/F_M_ was less affected (Fig. 1). At the protein level, the abundances of both photosystems were negatively affected (Fig. 2).

**Fig. 3:**
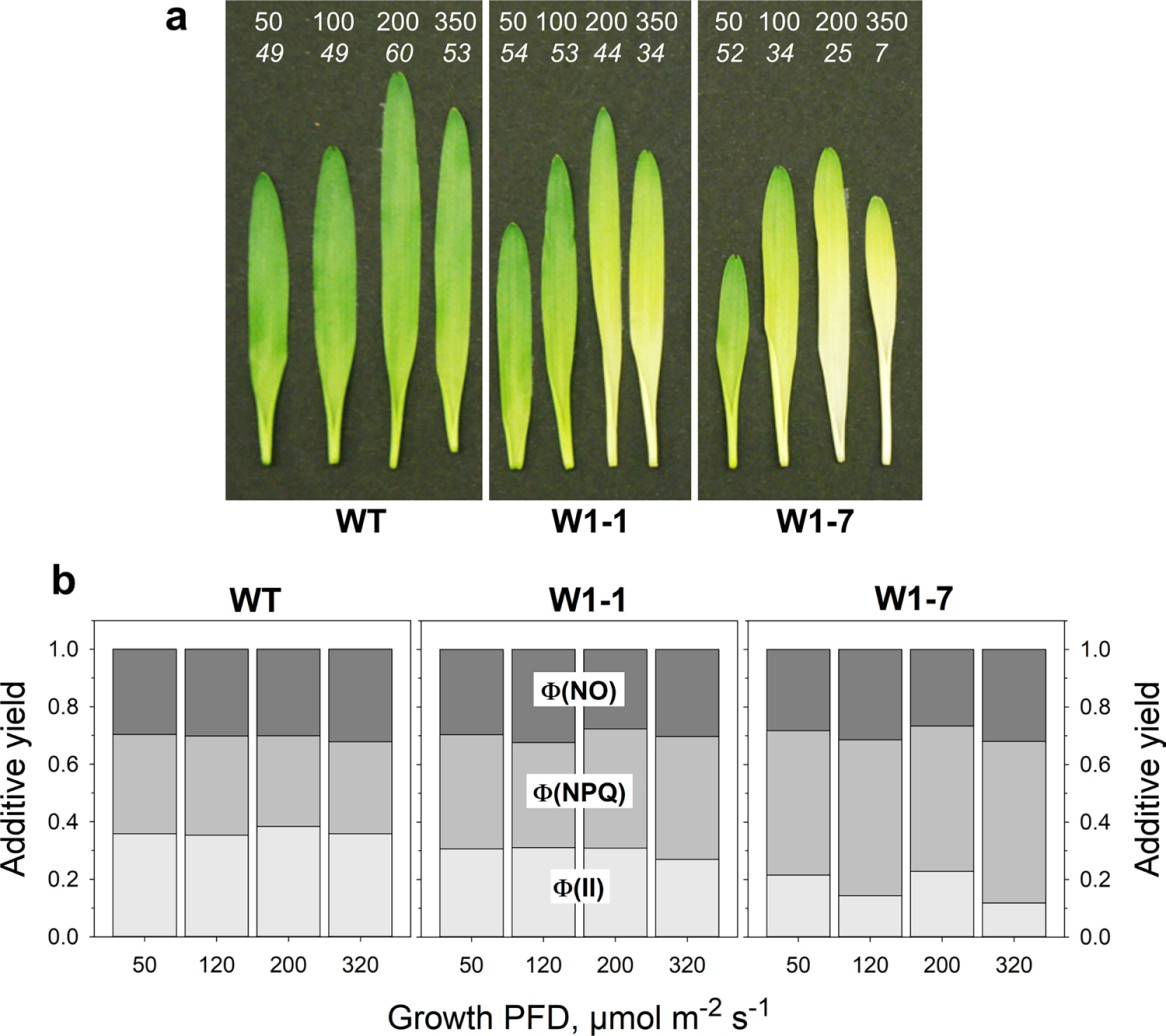
Characterization of primary foliage leaves collected at 10 das from barley wild type (WT), W1-1 and W1-7 lines grown in continuous light of four different irradiances. **a** Photographies illustrating the differences in chlorophyll contents (values from Swida-Barteczka et al. 2018), which are indicated on the top in italic numbers together with the irradiances during growth. **b** Fate of the light energy absorbed at PS II was determined at an irradiance of 340 µmol m^−2^ s^−1^. The quantum yield of photochemistry [$\Phi$ (II)], of regulated non-photochemical quenching [$\Phi$ (NPQ)] and nonregulated non-photochemical quenching [$\Phi$ (NO)] were calculated according to the formulas given in Materials and methods. Columns are means of *n* = 3 leaves. Results of two-way ANOVA are provided in Supplementary Table S2

In another previous study, it had been shown that leaves of the WHIRLY1-deficient plants became chlorotic when grown in continuous light and that the chlorosis got more vigorous with increasing irradiance coinciding with an increase in the level of ROS (Swida-Barteczka et al. 2018). To reinforce the differences observed in the WHIRLY1-deficient plants grown in a daily light–dark cycle, plants were grown in continuous light for the subsequent investigations. To begin with, primary foliage leaves of WT, W1-1, and W1-7 were collected after 10 days of growth in continuous light of four different irradiances and were photographed (Fig. 3a). As reported previously, the lengths of the W1 leaves were reduced in comparison to the wild type, whereby the W1-7 line having a lower level of WHIRLY1 than the line W1-1 (Krupinska et al. 2014) had the shortest leaves (Fig. 1a in Swida-Barteczka et al. 2018). As apparent in the photographs, leaves of all genotypes got longer with increasing irradiance except at the highest irradiance, which obviously caused photoinhibition indicated by the reduced F_V_/F_M_ (Swida-Barteczka et al. 2018). The chlorosis of leaves got stronger with increasing irradiance, and it was most pronounced in leaves of the W1-7 line (Fig. 3a). In line with the previous study, reporting an increased pool size of the violaxanthin cycle pigments (V, A, Z) and an increased de-epoxidation state in the W1 plants compared to the wild type (Swida-Barteczka et al. 2018), non-photochemical quenching (NPQ) increased with decreasing abundance of WHIRLY1 (Fig. 3b).

Chlorophyll fluorescence measurements on the fate of light energy absorbed by PSII showed that the quantum yield of PSII (Φ(II)) was reduced in the W1 lines. In contrast, nonregulated fluorescence (Φ(NO)) was not significantly different among the genotypes (Fig. 3b; two-way ANOVA, *P* = 0.128). On the other hand, significant differences between wild type and W1 lines regarding Φ(NPQ) were detected by two-way ANOVA (*P* < 0.001) (Supplementary Table S2). They were more pronounced for the W1-7 line than for the W1-1 line, where no significant difference was detected in comparison to the wild type at a growth irradiance of 50 µmol m^−2^ s^−1^. One may assume that the effect of WHIRLY1 deficiency on Φ(II) was compensated by a reciprocal effect on Φ(NPQ) (Saeid Nia et al. 2023).

#### Immunological detection of proteins of the photosynthetic apparatus

**Fig. 4:**
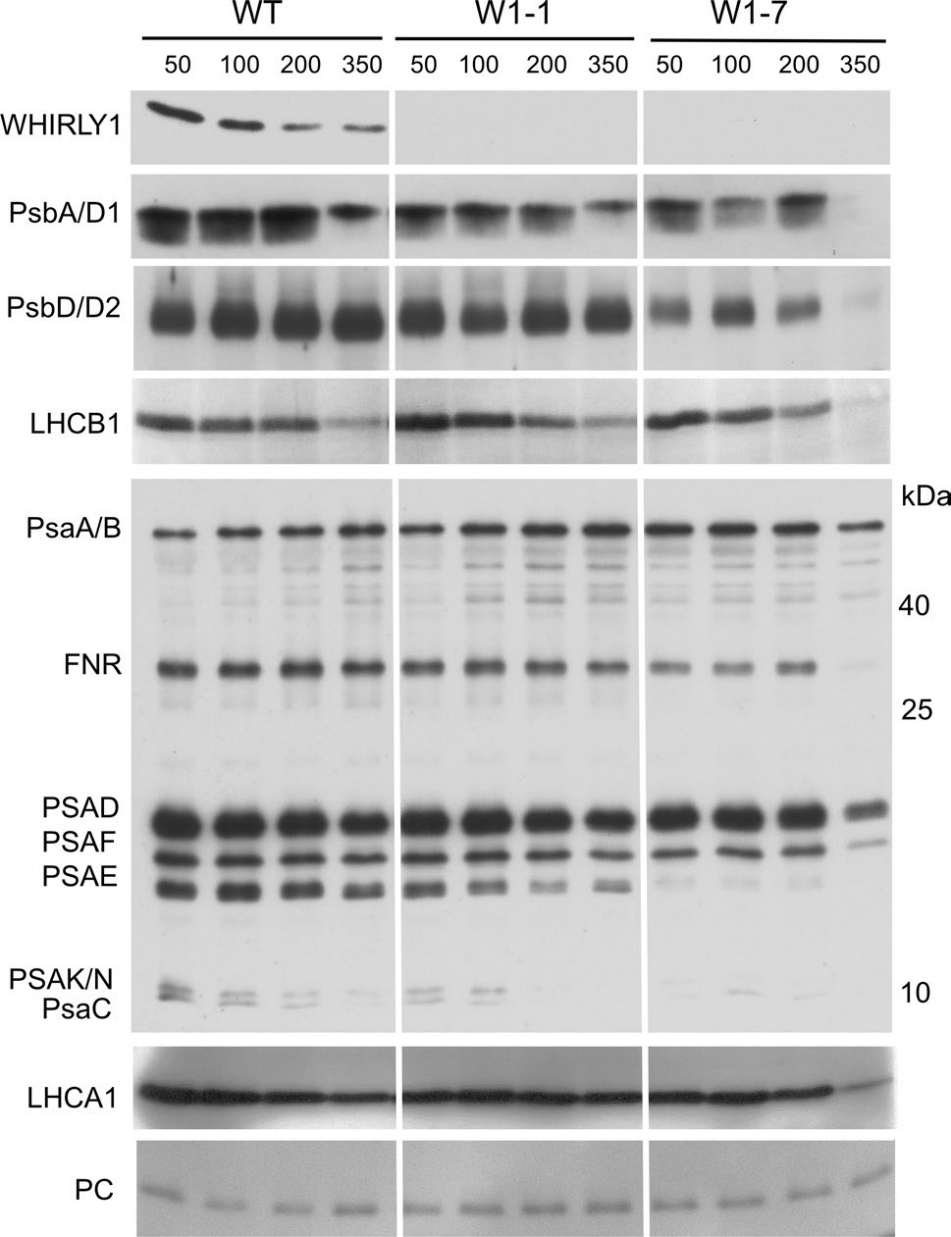
Immunoblot analyses of chloroplast proteins in primary foliage leaves of the wild type (WT) and the *WHIRLY1* knockdown lines W1-1 and W1-7. Immunoblots were performed with total leaf extracts from plants grown for 10 days in continuous light of four different irradiances (50, 100, 200, and 350 µmol m^−2^ s^−1^). Antibodies were directed against WHIRLY1, central proteins of PSII (PsbA, PsbD), LHCB1, PSI proteins, LHCA1, and plastocyanin (PC). The PSI proteins (PsaA/B, PSAD, F, E, K/N, and PsaC) and ferredoxin oxidoreductase (FNR) were detected by a complex antibody mixture recognizing several proteins of PSI. For visualization, immunoblots were exposed to film

Immunoblot analyses with fractions from the sucrose gradient showed reduced levels for several subunits of the photosynthetic apparatus in the W1-7 line (Fig. 2). To compare the levels more directly, gels used for immunoblotting were loaded with identical amounts of protein per lane. Specific individual antibodies were used to detect WHIRLY1, PSII reaction center proteins (PsbA/D1, PsbD), apoprotein LHCB1 of the major light-harvesting complex, the apoprotein LHCA1 of PSI associated light-harvesting complex, and plastocyanin. Several subunits of PSI were detected by a complex antibody fraction prepared against the entire PSI including the enzyme ferredoxin oxidoreductase (FNR) (Jensen et al. 2000). The immunological analyses showed that the abundance of light-harvesting complex proteins was rather stable in the three genotypes. In contrast, the amounts of reaction center proteins of PSII (PsbA, PsbD) were reduced in the W1-7 line. These data are in line with the findings obtained for plants grown in a daily light–dark cycle (Fig. 2). The reduction in the levels of reaction center proteins was most prominent after growth at high irradiance (Fig. 4). Among the PSI proteins, PSAE and two proteins, having molecular weights in the range of 8–10 kDa (PsaC, PSAK/N), had reduced levels in both W1-1 and W1-7 leaves, whereby their levels were lowest at the highest irradiance. Also, the strong signals detected on film for PsaA/B and PSAD were reduced in W1-7 at the highest irradiance. The complex antibody prepared against PSI also showed that the relative amount of ferredoxin oxidoreductase (FNR) depended on both the abundance of WHIRLY1 and irradiance. Also, its level was lowest in W1-7 leaves of plants grown at high irradiance (Fig. 4). In comparison, the abundance of the PSI electron donor plastocyanin was neither affected by WHIRLY1 abundance nor by irradiance.

#### The impact of WHIRLY1 on the accumulation of chloroplast superoxide dismutases at high irradiance

**Fig. 5:**
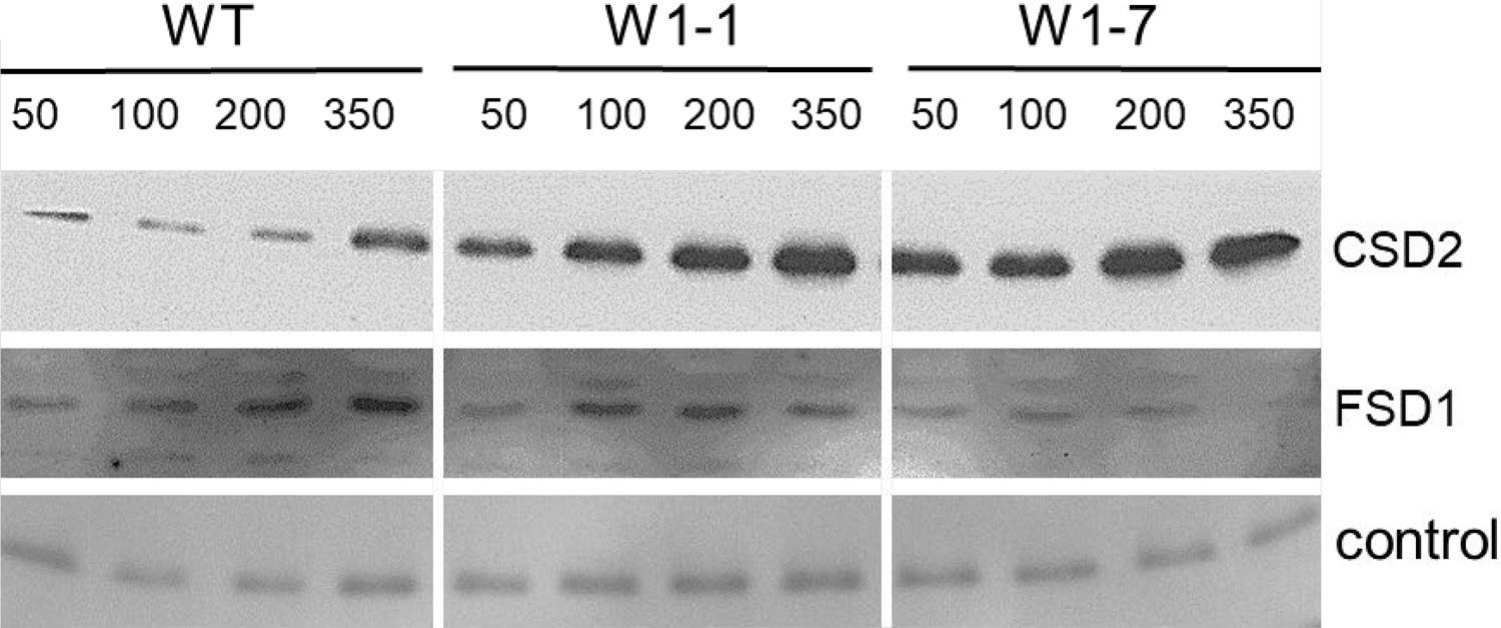
Immunological detection of chloroplast superoxide dismutases. Immunoblots were prepared with total proteins from primary foliage leaves of wild-type, W1-1 and W1-7 plants after growth in continuous light at different irradiances (50, 120, 200, and 350 µmol m^−2^ s^−1^). Specific antibodies were used to detect chloroplast CuZnSOD (CSD2) and FeSOD (FSD1). As a control for equal loading, the band detected by the antibody directed toward plastocyanin staying rather stable is shown (see also Fig. 4). For visualization, immunoblots were exposed to film

Previously, W1-1 and W1-7 seedlings were shown to accumulate ROS when grown in continuous light of high irradiance (Swida-Barteczka et al. 2018). The enhanced ROS levels could hint at a reduced antioxidative capacity of the chloroplasts. Chloroplasts have an iron-containing superoxide dismutase (FeSOD) besides a Cu/Zn-containing form (Cu/ZnSOD) for the removal of ROS. Immunoblot analyses with specific antibodies directed toward both types of chloroplast SODs showed that in the W1 lines at all four irradiances, the level of Cu/ZnSOD was much higher than in the wild type, whereby the level rose with increasing irradiance (Fig. 5). In comparison to the W1 lines, in the wild type, the level of Cu/ZnSOD increased only at the highest irradiance tested (Fig. 5). In contrast to Cu/ZnSOD, the level of the iron-containing FeSOD declined in leaves of the W1-7 line, while in leaves of the wild type, it increased with increasing irradiance, as expected (Fig. 5).

#### WHIRLY1-dependent accumulation of different classes of iron cofactor proteins

**Fig. 6:**
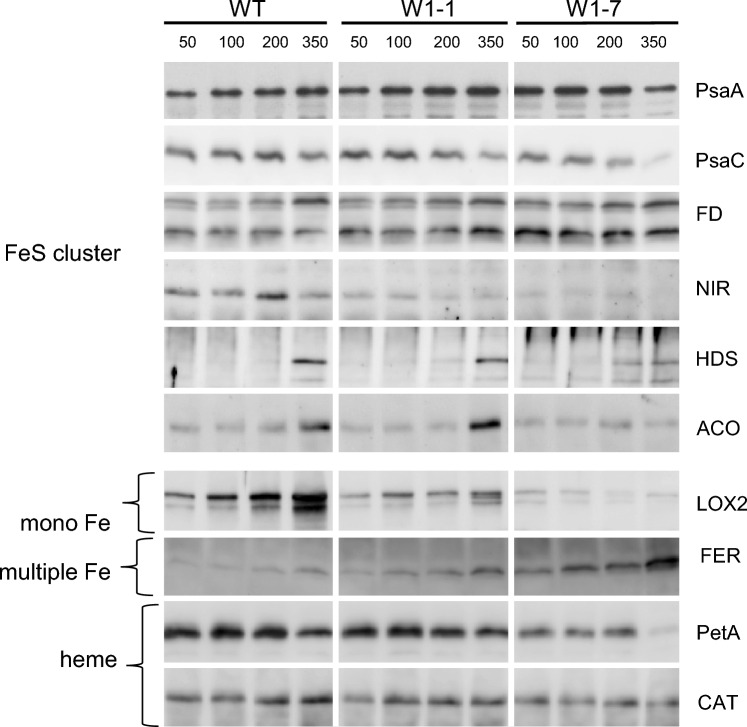
Immunoblot analyses of iron cofactor proteins in primary foliage leaves of the wild type (WT) and the *HvWHIRLY1* knockdown lines W1-1 and W1-7. Immunoblots were performed with total leaf extracts from plants grown for 10 days in continuous light of four different irradiances (50, 100, 200, and 350 µmol m^−2^ s^−1^). Antibodies were directed toward individual chloroplast proteins with iron–sulfur clusters, i.e., PsaA, PsaC, FD, NIR, HDS, and ACO. Additionally, LOX2 containing mononuclear iron, FER sequestering iron, and the heme-containing proteins PetA and CAT were detected. Most of these proteins are located in chloroplasts. CAT is a peroxisomal protein and ACO is located to mitochondria. *ACO* aconitase, *CAT* catalase, *FD* ferredoxin, *FER* ferritin, *HDS* hydroxymethyl butenyl diphosphate synthase, *LOX2* lipoxygenase 2, *NIR* nitrite reductase, *PetA* cytochrome f, *Psa* photosystem I core proteins. For visualization, immunoblots were evaluated with a ChemiDoc system allowing a quantification of signal intensities (Supplementary Fig. S6)

The differences observed in the function and composition of the photosynthetic apparatus as well as in the levels of the two chloroplast SODs indicate that in WHIRLY1-deficient plants in comparison to the wild type, iron may not be available in adequate amounts. The iron-containing proteins having reduced levels in the WHIRLY1-deficient plants have all different kinds of iron cofactors, i.e., heme, iron–sulfur clusters, or mononuclear iron as in case of FeSOD. This indicates that WHIRLY1 abundance affects all classes of iron proteins. To investigate this hypothesis, further chloroplast proteins having diverse iron cofactors were immunologically analyzed. In contrast to the immunoblots visualized by film (Figs. 4, 5), the immunoblots shown in Fig. 6 were visualized using a Bio-Imaging system allowing to quantify the signals (Supplementary Fig. S6).

For comparison with additional iron–sulfur cluster proteins, immunodetections of the two iron–sulfur cluster protein of PSI, PsaA (Fe_4_S_4_) and PsaC (2 × Fe_4_S_4_) are shown again in Fig. 6. In a different way as in Fig. 4, PsaC was detected using a specific antibody instead of the complex antibody mixture directed toward PSI. While the level of PsaA at the highest irradiance was reduced only in W1-7 leaves, the level of PsaC was reduced at this irradiance in both W1-1 and W1-7 leaves (Fig. 6, Supplementary Fig. S6). Another iron–sulfur protein functioning in photosynthesis is ferredoxin. Immunodetection with a specific antibody toward ferredoxin (Fe_2_S_2_) revealed that the levels of the two detectable forms of ferredoxin (Scheumann et al. 1998) were, however, not reduced in the W1 lines (Fig. 6).

A prominent iron–sulfur cluster containing chloroplast enzyme is nitrite reductase (NIR), in which a Fe_4_S_4_ cluster is coupled to a siroheme. Immunoblots revealed that its level was reduced in both W1-1 and W-7 leaves (Fig. 6, Supplementary Fig. S6). Irradiance had apparently only a little impact on its level. Another protein with a Fe_4_S_4_ cluster is the hydroxy-methylbutenyl diphosphate synthase (HDS/ISPG), an enzyme of the plastid non-mevalonate pathway for isoprenoid biosynthesis. Its level increased at the highest irradiance in wild-type and W1-1 plants and to a lesser extent in W1-7 plants (Fig. 6, Supplementary Fig. S6). The mitochondrial iron–sulfur cluster aconitase (Fe_4_S_4_), catalyzing the isomerization of citrate to isocitrate, had similar levels in the three genotypes at low irradiance, indicating that its level did not depend on WHIRLY1. In the wild type and in line W1-1, its level increased during growth at high irradiance. However, the light-dependent increase was not observed in line W1-7 (Fig. 6, Supplementary Fig. S6).

As reported above, the accumulation of iron superoxide dismutase (FeSOD/FSD1) at high irradiance was impaired in the W1 lines (Fig. 5). This enzyme contains a mononuclear iron cofactor. For comparison, the level of lipoxygenase (LOX) also having a mononuclear iron cofactor was investigated. The enzyme is located in chloroplasts, where it is involved in the formation of 12-oxo-phytodienoic acid (OPDA), the precursor of jasmonic acid. Its level dramatically increased in the wild type at high irradiance. At low irradiance, the LOX mRNA level was lower in WHIRLY1-deficient plants compared to wild-type plants. Its upregulation at high irradiance, as observed in the wild type, was much weaker in line W1-1 and did not occur in line W1-7 (Fig. 6). These findings suggest that a stress-induced increase in the amount of this enzyme depends on WHIRLY1 abundance. The results are in line with a trend to a reduced level of OPDA in the WHIRLY1-deficient lines (Supplementary Fig. S4).

A unique universal protein sequestering thousands of iron ions in its spheric cage-like oligomer is ferritin which in plants locates to chloroplasts (Liu and Theil 2005; Briat et al. 2010a, b). In contrast to the chloroplast iron enzymes hitherto analyzed, the level of ferritin was clearly higher in WHIRLY1-deficient lines and even increased at high irradiance (Fig. 6).

Finally, the levels of two enzymes containing heme cofactors were determined. One was chloroplast-located cytochrome f (PetA) and the other one was peroxisomal catalase (CAT). As already detected with the fractions from the sucrose gradients (Fig. 2b), the PetA level was lower in the W1-7 line, whereby its level was lowest at high irradiance (Fig. 6, Supplementary Fig. S6). In contrast to cytochrome f, the level of CAT located to peroxisomes was unaffected by WHIRLY1 abundance (Fig. 6, Supplementary Fig. S6).

Taken together, the levels of all iron-containing chloroplast proteins were lower in W1 leaves than in wild-type leaves except ferredoxin, whose level is known to be relatively stable under different conditions (Van Hoewyk et al. 2007), and ferritin whose level even increased in the W1 leaves. Despite these exceptions, the results indicate that the presence of WHIRLY1 affects the biosynthesis of the different types of iron cofactor-containing chloroplast proteins.

#### The concentration of iron and other heavy metals in primary foliage leaves of plants grown in continuous light of different irradiances

**Fig. 7:**
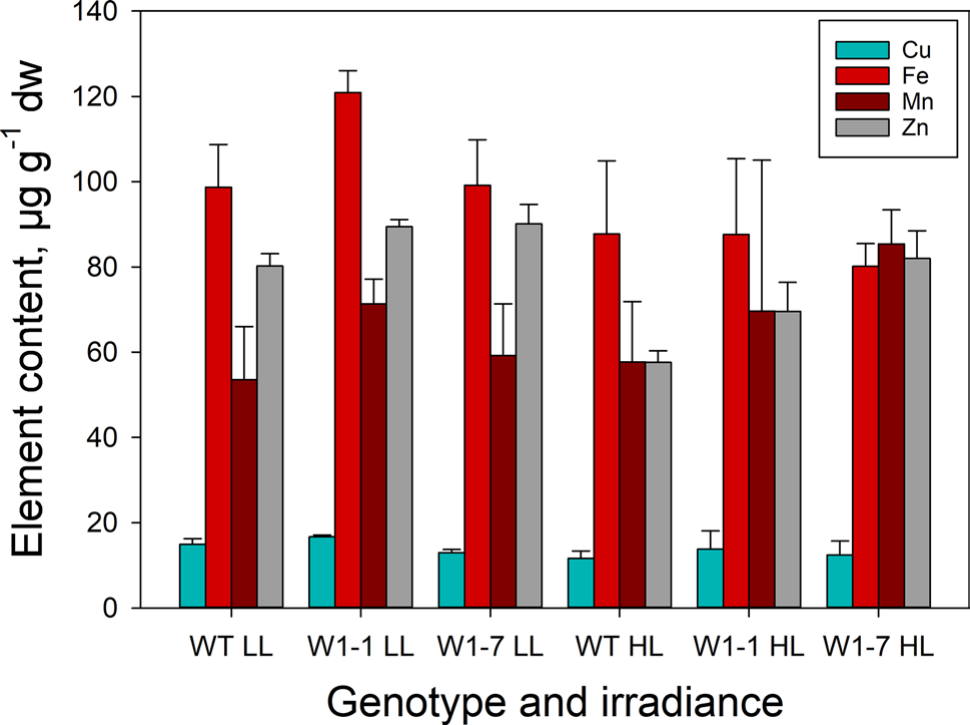
Metal content of leaves of the knockdown lines W1-1 and W1-7. The contents of copper, iron, manganese iron, and zinc were determined by mass ICP-mass spectrometry in desiccated primary foliage leaves collected from plants that were grown in continuous light of either 100 µmol m^−2^ s^−1^ (LL) or 350 µmol m^−2^ s.^−1^ (HL). Columns are means of three biological replicates +/− standard deviation

To determine whether differences in the concentration of iron and other heavy metals might be responsible for the differences in the altered abundances of iron-containing proteins and of iron and copper/zinc-containing chloroplast SODs between wild type and W1 lines, an inductively coupled plasma-mass spectroscopy (ICP-MS) (Jezek et al. 2015) analysis was performed with dried leaves, collected from plants that were grown at either an irradiance of 100 µmol m^−2^ s^−1^ (LL) or of 350 µmol m^−2^ s^−1^ (HL). Under both irradiances, no relevant differences were detected among the copper, iron, manganese, and zinc levels among the wild type and the W1 lines (Fig. 7). The low levels of iron-containing enzymes in WHIRLY1-deficient plants (W1) are not due to a low iron concentration in the leaves. The high level of ferritin in the W1 plants even indicates that chloroplasts of the WHIRLY1-deficient chloroplasts might contain an excess of iron being sequestered by ferritin to reduce oxidative stress (Briat et al. 2010a,b).

#### Expression of genes in WHIRLY1-deficient seedlings grown in continuous light of low and high irradiances

WHIRLY1 binds to DNA in both chloroplasts and the nucleus and might control the expression of genes encoding photosynthesis-associated proteins in both compartments as well as nuclear genes encoding the iron cofactor chloroplast enzymes (Krupinska et al. 2024). To investigate whether changes in gene expression contribute to the WHIRLY1-dependent differences in protein levels, mRNA levels of genes encoding photosynthesis-associated genes and iron-containing enzymes were analyzed in primary foliage leaves of plants grown at either 100 or 350 µmol m^−2^ s^−1^ (LL, HL).

**Fig. 8:**
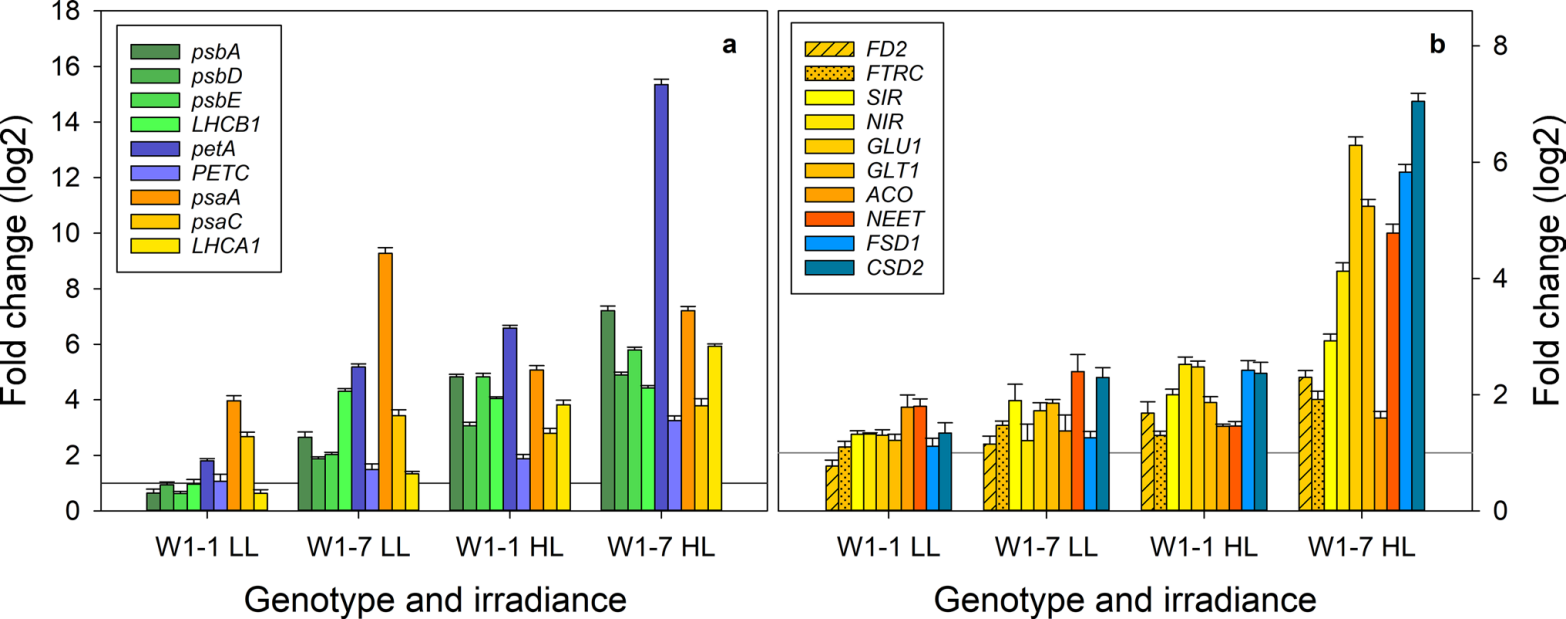
Gene expression in primary foliage leaves of wild-type (WT), W1-1, and W1-7 seedlings grown in continuous light of low (LL: 100 µmol m^−2^ s^−1^) or high irradiance (HL: 350 µmol m^−2^ s^−1^). **a** Expression of genes encoding photosynthesis-associated proteins: genes for PSII center proteins (*psbA, psbD*) cytochrome f (*petA*), the Rieske protein of the cytochrome b_6_f complex (*PETC*), subunits of PSI (*psaA, psaC*), and subunits of the light-harvesting complexes (*LHCB1* and *LHCA1*), **b** Expression of genes encoding chloroplast iron–sulfur cluster proteins (yellow and orange) are *FD2, FTRC, SIR*, *NIR, GLU1, GLT1, ACO,* and *NEET*. *FSD1* and *CSD2* (blue) encode chloroplast superoxide dismutases. Expression levels were calculated relative to the expression levels in the respective wild type at LL and HL. Primers are listed in the Supplementary Table S1. *ACO* aconitase, *CSD2* Cu/Zn superoxide dismutase, *FD2* ferredoxin, *FSD1* Fe superoxide dismutase, *FTRC* ferredoxin thioredoxin reductase, *GLT1* glutamate synthase, *GLU1* glutamine synthase, *NEET* iron–sulfur transfer protein, *NIR* nitrite reductase, *SIR* sulfite synthase

At low light, genes encoding PsaA, PsaC, and cytochrome f (PetA) had elevated expression levels in the W1 lines compared to the wild type. At high irradiance, mRNA levels of all genes encoding PSII core components (*psbA, psbD, psbE*), cytochrome f (*petA*), and the genes encoding PSI core components (*psaA* and *psaC*) were elevated in both W1 lines in comparison to either the wild-type data at two irradiances, set to 1, respectively (Fig. 8), or to the wild-type data at low irradiance (Supplementary Fig. S5). The highest level was detected for the *psaA* mRNA, which in the W1-7 line had a ninefold higher level than in the wild type (Fig. 8a, Supplementary Fig. S5a). While expression of the nuclear gene *LHCB1* was not altered in the W1-1 line, its mRNA had a fourfold higher level in the W1-7 line. At high irradiance, the expression levels of all genes tested were higher in both W1 lines compared to the wild type. The enhanced expression of the genes could be partly due to ROS, whose levels were higher in W1-1 and W1-7 than in the wild type (Swida-Barteczka et al. 2018). The highest expression level at high irradiance (15-fold in comparison to the wild type) was detected for *petA* in the W1-7 line (Fig. 8a). For comparison, the expression level of the nuclear-encoded Rieske protein (PETC) was only less than fourfold enhanced (Fig. 8a)*.*

Immunoblot analyses did reveal that besides iron cofactor proteins of the photosynthetic apparatus (PsaA, PsaC, and PetA), several other iron cofactor chloroplast proteins without a direct function in photosynthesis had lower abundances in WHIRLY1 deficient plants (Fig. 6). qRT-PCR was used to investigate whether the mRNA levels of the corresponding genes show concurrent changes as the proteins (Fig. 8b).

Under low irradiance, the levels of all mRNAs encoding iron–sulfur proteins (FD2, FTRC, SIR, NEET, NIR, GLU1, GLT1, and ACO) barely differed between W1 lines and wild type. At high irradiance, expression levels of all genes except *ACO* increased in the WHIRLY1-deficient lines, whereby the mRNA levels of *NIR, GLU1, GTL1, ACO, and NEET* increased about five-to-sevenfold in the W1-7 line compared to the wild type (Fig. 8b). The mRNA levels of *NIR* and *ACO* are not in line with the reduced levels of the corresponding proteins in WHIRLY1-deficient plants (Fig. 6), indicating that the low abundance of these proteins in the WHIRLY1-deficient lines is not regulated at the level of mRNA.

To investigate whether the SOD protein levels (Fig. 5) are regulated by gene expression, also the mRNA levels of the two chloroplast-located forms of SOD were compared. In line with the high protein level, the expression of *CSD2* encoding Cu/ZnSOD was enhanced already at an irradiance of 100 µmol m^−2^ s^−1^ (Fig. 8b). Although the FeSOD protein level of WHIRLY1 deficient plants did not increase at an irradiance of 350 µmol m^−2^ s^−1^ (Fig. 5), the *FSD1* mRNA level increased almost as much as that of *CSD2* in both W1 lines (Fig. 8), indicating that the reduced protein level of the FSD1 protein in W1 plants is not regulated at the level of mRNA.

Regarding the toxic effects of excess iron, its uptake, transport, distribution, and storage need to be tightly controlled. Numerous proteins are involved in the maintenance of iron homeostasis in chloroplasts. To get insight into the consequences of the reduced iron bioavailability in WHIRLY1-deficient chloroplasts, additionally, the expression of few key components of iron homeostasis was investigated.

**Fig. 9:**
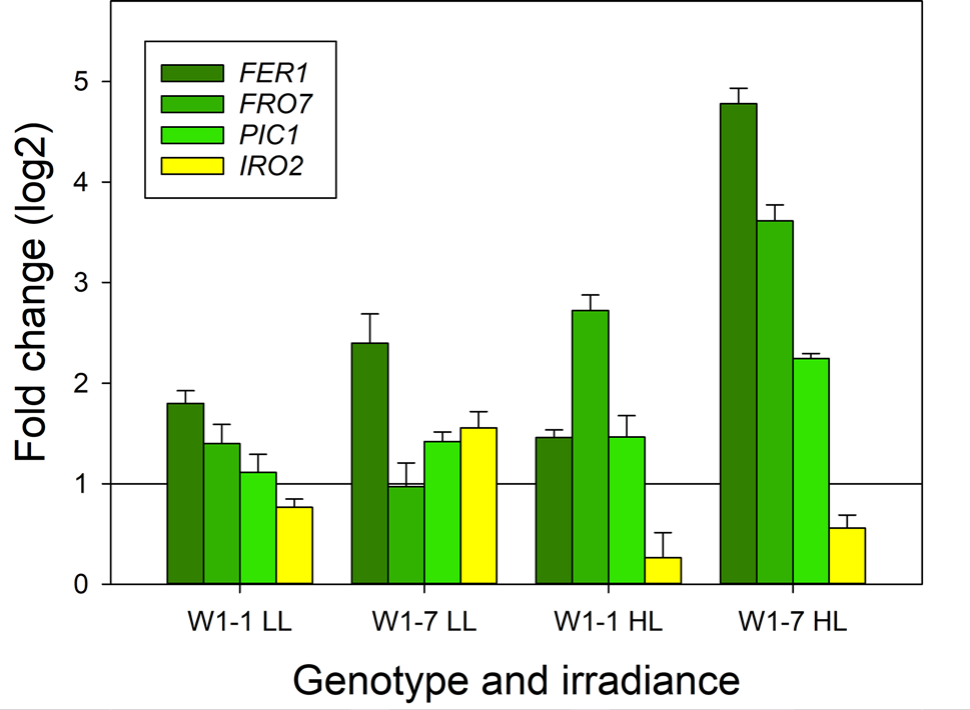
Expression of genes encoding proteins involved in iron homeostasis. RNA was extracted from primary foliage leaves of wild-type (WT), W1-1 and W1-7 seedlings grown in continuous light of low (LL: 100 µmol m^−2^ s^−1^) or high irradiance (HL: 350 µmol m^−2^ s.^−1^). Primers are listed in the Supplementary Table S1. *FER1* ferritin, *FRO7* ferric chelate reductase; *IRO2* iron-regulated bHLH transcription factor, *PIC1 PERMEASE IN CHLOROPLASTS 1*

To begin with the expression of a master regulator of iron uptake was investigated. Iron uptake from the soil is regulated by a transcription factor cascade consisting of bHLH family transcription factors (Riaz and Guerinot 2021) being upregulated under iron deficiency (Kroh and Pilon 2020). In rice, IRO2 is the master regulator of the chelation-based uptake of iron (Wang et al. 2020). In primary foliage leaves from WHIRLY1-deficient plants grown in continuous light of 350 µmol m^−2^ s^−1^ (HL), the expression of *IRO2* was reduced in comparison to wild-type leaves, indicating that these leaves do not lack iron, but rather have an excess of free iron (Fig. 9).

Although the mechanism of iron acquisition by chloroplasts is still not fully understood, the two proteins FRO7 and PIC1 were shown to be important in this process (Przybyla-Toscano et al. 2018; Vigani et al. 2019). FRO7 mediates the reduction of ferric iron to ferrous iron in the intermembrane space. PIC1 (PERMEASE IN CHLOROPLASTS 1), a component of the protein import machinery of the inner envelope, was suggested to act as a permease for iron (Duy et al. 2007). Expression of both, *FRO7* and *PIC1,* was enhanced in primary foliage leaves of WHIRLY1-deficient lines W1-1 and W1-7 grown in continuous light of 350 µmol m^−2^ s^−1^. Only *PIC1* expression was slightly enhanced in line W1-7 at 100 µmol m^−2^ s^−1^ (Fig. 9).

Whereas in the case of the iron proteins PsaA/C, NIR, ACO, and PetA, the levels of mRNAs (Fig. 8) did not parallel the levels of the corresponding proteins (Fig. 6), in the case of ferritin, both the protein level (Fig. 6) and the mRNA level increased in the WHIRLY1-deficient line W1-7 (Fig. 9). The level of FER1 mRNA increased more than twofold at low irradiance and almost fivefold at high irradiance, indicating that the deficiency of WHIRLY1 is a condition stimulating *FER1* expression (Fig. 9).

## Discussion

Considering that the WHIRLY1-deficient barley plants (W1-1, W1-7) display delayed chloroplast development and become chlorotic under conditions of high irradiance (Krupinska et al. 2019; Swida-Barteczka et al. 2018; Saeid Nia et al. 2022), this study addressed the question whether they might suffer from iron deficiency. However, the measurement of leaf metal concentrations revealed that leaves of the WHIRLY1 deficient lines had a regular iron content. Nevertheless, the alterations observed in the function and composition of the photosynthetic apparatus of W1 lines are comparable to those observed in plants grown under iron deficiency conditions as outlined in the following.

### The photosynthetic apparatus of WHIRLY1-deficient plants resembles that of iron-deficient plants

The photosynthetic apparatus has a high demand for iron (Briat et al. 2015). Therefore, PSI contains most iron atoms, and, accordingly, a limitation of this element destabilizes the photosystem (Briat et al. 2015). Among the iron-containing proteins of PSI are the reaction center proteins PsaA and PsaB, both having iron–sulfur cluster cofactors (Fe_4_S_4_) that mediate charge separation and electron transfer. Most of the iron in PSI is contained in PsaC which has two iron–sulfur clusters of the Fe_4_S_4_ type. The immunological analyses showed that the level of PsaA was relatively stable in all genotypes. In contrast, the levels of PsaC, PSAE, and small molecular weight proteins (one of them, likely representing PSAK) strongly decreased in the W1 lines at all conditions whereby the decrease was most pronounced in the W1-7 line grown in continuous light of the highest irradiance applied (Fig. 4). The high stability of the reaction center proteins PsaA/B could be due to their prioritized supply with iron (Moseley et al. 2002). PsaC has a function in stabilizing PSI besides its role in electron transport. Insertional inactivation of *psaC* in *Chlamydomonas* led to a destabilization of the photosystem as evidenced by the reduced levels of further PSI proteins in the mutant (Takahashi et al. 1991). Although PSAE and PSAK do not contain iron, their levels are also reduced under iron deficiency, indicating that the complete photosystem is remodeled during this situation (Moseley et al. 2002). PSAE is a docking protein for the Fe_2_S_2_ containing ferredoxin mediating electron transfer behind PSI (Jensen et al. 2007). Arabidopsis plants with a *PSAE* knockdown (Scheller et al. 2001) or a mutation of the gene (Varotto et al. 2000) were pale and highly susceptible to photoinhibition, suggesting that the protein might be necessary for the stability of the complex besides its role in binding to ferredoxin and its involvement in cyclic electron flow (Scheller et al. 2001). A decrease of PSAK in *Chlamydomonas* grown at low iron concentrations was shown to destabilize the linkage between LHCI and the PSI core complex, thereby minimizing photooxidative damage (Jensen et al. 2000; Moseley et al. 2002). The reduced PSAK level in WHIRLY1-deficient plants is likely the reason for the high abundance of LHCA1 disconnected from the PSI holocomplex in sucrose gradients (Fig. 2b) and on clear native gels (Fig. S2) coinciding with the shift of the long-wavelength fluorescence emitted by LHCA to shorter wavelengths as detected at 77 K (Fig. S2).

Disconnection of the LHCA complex from the photosystem I core complex has been proposed to be an early response to a reduced iron supply in chloroplasts which immediately leads to a decline in energy flow to the reaction centers, thereby reducing photoinhibition of the photosynthetic apparatus (Moseley et al. 2002). Remodeling of the antenna system of the photosynthetic apparatus as a mechanism lowering light absorption and favoring energy-dissipative processes involves besides LHCA also the LHCB complex (Moseley et al. 2002; Larbi et al. 2006) which is predominantly associated with PSII. While the level of LHCA1 mRNA was not altered in WHIRLY1-deficient plants grown at low irradiance, the mRNA level of LHCB1 was increased in the W1-7 line (Fig. 8). An enhanced expression of *LHCB1* as observed for the W1 lines (Fig. 8) is typical for prolonged iron deficiency (Saito et al. 2010). On immunoblots with samples having the same amount of protein, the level of the LHCB1 protein was rather stable at low irradiance and only declined in the W1-7 line at highest irradiance (Fig. 4). LHCB1 has been reported to contribute to photoprotection in iron-deficient barley by its function in thermal dissipation (Saito et al. 2010). The stable LHCB1 level of the WHIRLY1-deficient plants is in line with the enhanced NPQ. A high NPQ in W1-7 leaves was proposed to contribute to the survival of W1 plants under oxidative stress conditions (Saeid Nia et al. 2023).

In comparison to LHCB1, the PSII reaction center proteins had much lower levels in the W1-7 line (Fig. 4). Reduced levels of PSII center proteins have also been observed in conditions of iron deficiency (Hantzis et al. 2018). Indeed, also PSII depends on the availability of iron. The heme-containing cytochrome b559 has been proposed to play a role in the assembly of PSII (Chu and Chiu 2016). A non-heme iron protein (NHI) has been shown to be required for electron transfer at the acceptor side (Müh and Zouni 2013). Alterations in the organization of PSII under iron deficiency conditions have been linked to enhanced dissipation of excitation energy (Abadia et al. 1999) as a means to diminish oxidative damage.

Electron transport from PSII to PSI is mediated by the cytochrome b_6_f complex. The complex contains iron in the form of heme, which is a cofactor of cytochromes, and in the form of a Fe_2_S_2_ cluster being a cofactor of the Rieske protein (PETC). A diminished abundance of the complex in WHIRLY1-deficient plants is evident by a reduction in the level of cytochrome f in the sucrose gradient fractions obtained with thylakoid membranes of the line W1-7 (PetA, Fig. 2). This finding is in line with the low absorbance change measured for plastocyanin (Supplementary Fig. S1) indicating a reduced electron flow from PSII to PSI.

Taken together, the photosynthetic apparatus of WHIRLY1-deficient plants resembles that of iron-deficient plants, regarding both composition and functionalities of the photosystems, even though the iron content of the WHIRLY1 deficient leaves is not lower than that of the wild-type leaves. The enhanced mRNA levels measured for plastid-encoded proteins of the photosystems and the cytochrome b_6_f complex (PsbA, PsbD, PsbE, PsaA*,* PsaC, and PetA) and the mRNA levels of nucleus-encoded components of the thylakoid membrane proteins (LHCA1, LHCB1, and PETC) indicate that WHIRLY1 affects the abundances of the photosynthesis-associated proteins not at the level of mRNA, but rather at the post-transcriptional level.

### WHIRLY1 plays a role in the assembly of different classes of iron proteins in chloroplasts

Illuminated chloroplasts from WHIRLY1-deficient plants were shown to produce more ROS than wild-type chloroplasts (Swida-Barteczka et al. 2018). It was, therefore, evident to compare the levels of the two superoxide dismutases in chloroplasts. One of them has a mononuclear iron as a cofactor (FeSOD/FSD1), and the second instead has copper and zinc as cofactors (CuZnSOD/CSD2). The immunological analyses showed that in all genotypes, the level of CuZnSOD increased with increasing irradiance, whereas the level of FeSOD only increased in the wild type. The results indicate that in WHIRLY1-deficient plants, iron is not available for the stress-induced upregulation of iron-containing superoxide dismutase. While the levels of the two enzymes responded differently to irradiance in the WHIRLY1-deficient plants, mRNA levels for both enzymes increased concomitantly at high irradiance, indicating that the FeSOD protein level is not regulated at the level of mRNA but likely depends on the availability of iron (Shams et al. 2024).

Taken together, the immunological analyses on the composition of the photosynthetic apparatus and the levels of chloroplast SODs indicate that WHIRLY1 plays a role in the formation of different forms of iron cofactors, i.e., Fe–S cluster, heme, and non-Fe–S/non-heme iron cofactors (Przybyla-Toscano et al. 2021). To investigate whether WHIRLY1 has a general impact on iron-containing proteins, the levels of further iron cofactor proteins have been immunologically determined.

Additional chloroplast proteins with Fe–S clusters tested were ferredoxin (FD) and hydroxy-3-metylbutene diphosphatase (HDS), an enzyme of the non-mevalonate isoprenoid biosynthesis. For comparison, the level of the mitochondrial Fe–S cluster enzyme aconitase (ACO) was analyzed. Surprisingly, the ferredoxin (FD) level was neither affected by WHIRLY1 abundance nor light. An extraordinarily high stability of FD has even been observed in plants with a limiting iron–sulfur cluster biosynthesis induced by the knockdown of the *NFS/NifS* gene (Van Hoewyk et al. 2007). At low irradiance, the level of HDS was almost undetectable. At the highest irradiance, it increased in all genotypes whereby the increase was lowest in the W1-7 line being in line with a promoting impact of WHIRLY1 on the formation of HDS. The level of mitochondrial aconitase did not respond to WHIRLY1 abundance at low irradiance, indicating that WHIRLY1 has no impact on mitochondrial Fe–S cluster protein formation. Surprisingly, the aconitase level was upregulated at high irradiance in the wild type and in W1-1 plants, but not in W1-7 plants. Its upregulation in response to high irradiance could be either caused by ROS accumulating in the WHIRLY1-deficient chloroplasts or mediated by the deficiency of WHIRLY1 itself which can regulate genes also negatively (Krupinska et al. 2022).

Another chloroplast enzyme tested was nitrite reductase (NIR) containing a Fe_4_S_4_ cluster connected with a siroheme cofactor (Balk and Schaedler 2014). While FD requires only two iron per protein molecule, NIR requires five iron atoms per protein. Its level correlated with the abundance of WHIRLY1 having the lowest abundance in the W1–7 line. In contrast to HDS and ACO, the level of NIR did not depend on the irradiance during growth.

Furthermore, the levels of two heme-containing proteins were tested with the extracts from primary foliage leaves of the three genotypes grown at different irradiances. In line with the results of the sucrose gradient analysis (Fig. 2), the level of PetA was reduced in samples from the WHIRLY1-deficient plants. In contrast, the level of peroxisomal catalase (CAT) was not affected, although in plants heme is produced in chloroplasts (Tanaka and Tanaka 2007; Yurina et al. 2012) and, from there, also provided to peroxisomes. The stable level of CAT indicates that WHIRLY1 does not have a direct effect on heme biosynthesis.

For comparison with the mononuclear iron-containing FeSOD, a second chloroplast protein with a mononuclear iron cofactor was tested, i.e., lipoxygenase 2 (LOX2) which catalyzes the oxygenation of α-linolenic acid followed by enzymatic steps leading among others to OPDA, the chloroplast-located precursor of jasmonic acid (Wasternack and Song 2017). It is obvious that both its level at low irradiance and its light-dependent upregulation depend on WHIRLY1 (Fig. 6).

Apparently, chloroplast proteins with various forms of iron cofactors (Przybyla-Toscano et al. 2021) are downregulated in WHIRLY1 deficient chloroplasts. Chloroplasts are independent of cofactor supply from another cellular compartment. They contain their own Fe–S cluster assembly machinery of prokaryotic origin called SUF (SULFUR mobilization), consisting of six proteins involved in the desulfuration of cysteine and delivery of Fe–S clusters to recipient proteins (Balk and Schaedler 2014; Connorton et al. 2017). The SUFE3 component itself is a Fe–S cluster protein. Recently, two DnaJ proteins binding iron via cysteine residues were shown to interact with the SUF machinery to facilitate iron utilization for chloroplast Fe–S cluster biogenesis (Zhang et al. 2021). Although the DnaJ proteins were identified recently as iron donors of the SUF machinery, it is unknown how iron is provided to these proteins and the other iron proteins in which the iron is coordinated by amino acids, such as cysteine, aspartate, histidine, glutamate, serine, and arginine (Balk and Schaedler 2014; Przybyla-Toscano et al. 2021).

The specific changes observed in the photosynthetic apparatus of the WHIRLY1-deficient plants and the concomitant decreases in the levels of other iron–sulfur cluster containing chloroplast proteins resemble the changes reported for Arabidopsis plants with an induced knockdown of the gene encoding the plastidic cysteine desulfurase NFS2 also called CpNifS (Van Hoewyk et al. 2007; Balk and Pilon 2011). W1 plants as well as the plants with a knockdown of *NSF2* (conditional loss-of-function via RNAi for *NDF2*) displayed defects in photosynthetic electron transport, which can be ascribed to a defect in PSI and decreased levels of chloroplast proteins with different types of iron–sulfur clusters (Van Hoewyk et al. 2007). Despite these similarities, the impact of WHIRLY1 on iron homeostasis is more general affecting the allocation of iron for all kinds of iron cofactor proteins of chloroplasts.

In contrast to the iron-containing proteins whose levels were lower in WHIRLY1-deficient plants, the level of ferritin correlated inversely with the abundance of WHIRLY1. Ferritins are universal 24-meric proteins which store up to 4500 iron atoms inside their spherical cage-like oligomeric structure, and were shown to provide iron for metabolism in mammals (Harrison and Arosio 1996; Theil et al. 2006). In analogy, chloroplast-located ferritin would be the obvious candidate to provide iron for the biosynthesis of iron proteins during chloroplast development (Balk and Schädler 2014). However, mutants with mutations in the three ferritin genes of Arabidopsis were not impaired in chloroplast development, but had higher levels of ROS and increased levels of enzymes involved in detoxification (Ravet et al. 2009). It has been concluded that iron storage by ferritin in plants is not essential for producing iron cofactor proteins during chloroplast development. Instead, ferritins act as scavengers of free iron, thereby preventing oxidative stress (Briat 1996; Ravet et al. 2009).

Irradiance-dependent upregulation of the ferritin protein level was observed in both wild type and the W1 lines, whereby upregulation was much higher in the W1 lines. The stability of photosynthesis-associated protein complexes is likely diminished in WHIRLY1-deficient chloroplasts leading to the release of iron. The light-dependent accumulation of ferritin in WHIRLY1 deficient plants suggests that, at least partly, WHIRLY1 shares functions with ferritin and that the increased abundance of ferritin might compensate for the reduction in the level of WHIRLY1.

### Impact of WHIRLY1 deficiency on gene expression

Under iron deficiency, the genetic program is remodeled. The reprogramming of gene expression includes the downregulation of genes encoding photosynthesis proteins, being in line with the high demand of the photosynthetic machinery for iron (Kroh and Pilon 2020). Conversely, in the WHIRLY1-deficient plants, both nucleus and plastid-encoded genes encoding proteins of the photosynthetic apparatus are upregulated, likely reflecting a demand for the formation of these proteins or the assembly of thylakoid membrane protein complexes, which consist of both plastid and nuclear-encoded components.

In line with the enhanced expression of photosynthesis-associated genes, expression of the gene encoding the master transcription factor IRO2 is vice versa downregulated. Under iron deficiency, expression of *IRO2* and further genes encoding members of the bHLH transcription factor family are upregulated to enhance iron uptake (Riaz and Guerinot 2021). The concurrent increase in the *FER1* mRNA level at high irradiance aligns with its transcriptional upregulation by free iron (Arnaud et al. 2006; Briat et al. 2010b). Free iron is toxic, because it can react with oxygen to produce ROS such as the very reactive hydroxyl radicals in the so-called Fenton reaction (Meneghini 1997). The enhanced levels of ferritin protein and *FER1* mRNA in W1 lines compared to the wild type accordingly align with the accumulation of ROS in chloroplasts of W1 plants grown in continuous light of high irradiance (Swida-Barteczka et al. 2018).

Expression of *FRO7* and *PIC1* do not react to iron abundance (Mukherjee et al. 2006; Duy et al. 2007). An enhanced expression of *FRO7* and *PIC1* could indicate a demand for iron required for the formation of iron proteins, which was shown to be impaired in the WHIRLY1-deficient plants (Figs. 5, 6).

Taking together, the results on gene expression underline that chloroplasts of WHIRLY1 deficient plants do not suffer from iron deficiency, but rather contain an excess of iron, whose availability for protein synthesis and formation of thylakoid membrane complexes is, however, diminished.

### Proposed role of WHIRLY1 in iron homeostasis

Although the iron concentration of WHIRLY1-deficient leaves is in the same range as in wild-type leaves, iron transfer to proteins is impaired. Considering that WHIRLY1 abundance affects not a specific type of iron protein, but the formation of diverse iron cofactor proteins, it is likely that WHIRLY1 has a general role in iron homeostasis.

WHIRLY1 is the major architectural protein of chloroplast nucleoids which serve as a platform for the formation of ribosomes (Bohne et al. 2014). Accordingly, ribosomes are diminished in the WHIRLY1-deficient barley plants (Krupinska et al. 2019). In addition, various other protein complexes required for chloroplast development have low abundances in chloroplasts lacking WHIRLY1 (Krupinska et al. 2024). However, WHIRLY1 deficiency affected not only plastid-encoded gene products, but also nucleus-encoded proteins. While protein levels were reduced, expression of the corresponding genes was enhanced in conditions of WHIRLY1 deficiency. This indicates that limited assembly of chloroplast proteins rather than reduced translation may be the reason for impaired protein formation in the WHIRLY1-deficient plants.

Regarding the intensification of the phenotype of WHIRLY1-deficient plants under high irradiance conditions, the formation of ROS likely adds to the phenotype (Swida-Barteczka et al. 2018). In the WHIRLY1-deficient chloroplasts, ROS may be formed by iron released from degraded complexes of the photosynthetic apparatus. This assumption is in line with the irradiance-dependent increase of the abundance of the ferritin protein and *FER1* expression known to respond to free iron (Briat et al. 2010b).

Intriguingly, the structure of oligomeric WHIRLY1 resembles that of ferritins (Cappadocia et al. 2010, 2012) and that of the bacterial DNA-binding protein Dps (DNA-binding protein of starved cells) (Calhoun and Kwon 2011), which both are known for their iron-storing capacities and their antioxidative ferroxidase activities (Briat et al. 2010b; Zhao et al. 2002). WHIRLIES share with these proteins the formation of hollow spheres which consist of 24 monomers in the case of ferritins and WHIRLIES and 12 monomers in the case of Dps, the latter also being named mini-ferritin (Haikarainen and Papageorgiou 2010). The oligomeric forms of ferritin and Dps enclose a cage-like nanocavity which can store thousands of iron atoms (Grant et al. 1998; Liu and Theil 2005). Although it remains to be determined whether WHIRLY oligomers contain iron, the structural similarities with the iron-storing oligomeric complexes of Dps and ferritins suggest overlapping functions. This idea is also supported by the finding, that the level of the ferritin protein together with the expression of *FER1* is upregulated in WHIRLY1-deficient lines, suggesting that ferritin might partly compensate for WHIRLY1 deficiency. However, opposite to the lack of ferritin, WHIRLY1 deficiency has a negative impact on chloroplast development. Unlike chloroplast ferritin, WHIRLY1 might not only sequester iron, but also provide it for protein formation.

WHIRLIES, Dps, and ferritins likely share functions in protection against oxidative stress. So far, it remains unknown whether WHIRLIES have ferroxidase activity as shown for Dps and ferritin. In contrast to ferritins, Dps and WHIRLIES belong to the so-called nucleoid-associated proteins (NAP), which bind unspecifically to DNA and determine the architecture of nucleoids in bacteria and plant organelles. For Dps it has been shown that its nucleoid shaping activity is an important mechanism to protect DNA during stress conditions (Holowka and Zakrzewska-Czewinska 2020). It is striking that both WHIRLY1 and Dps, in addition to their DNA-associated role, share functions in iron homeostasis, indicating a conserved link between iron and nucleoid organization. Indeed, oxidative DNA damage is induced by free iron (Meneghini 1997). NAP-dependent remodeling of nucleoids during stress is a means to prevent oxidative base modifications and formation of DNA breaks, being in line with the proposed role of WHIRLY proteins in maintenance of plant organelle genome stability (Marechal et al. 2009; Marechal and Brisson 2010). Considering that all WHIRLIES share the sphere-like oligomeric structure, WHIRLY2 might have a similar function in mitochondria (Negroni et al. 2024) as WHIRLY1 in chloroplasts.

## Supplementary Information

Below is the link to the electronic supplementary material.

Supplementary file1 (PDF 496 KB)

Supplementary file2 (PDF 472 KB)

Supplementary file3 (PPTX 120 KB)

Supplementary file4 (DOCX 21 KB)

Supplementary file5 (DOCX 14 KB)

## Data Availability

All data supporting the findings of this study are available in the manuscript and in its Supplementary Information.

## Abbreviations

das: Days after sowing
Dps: DNA-binding protein of starved cells
LHC: Light harvesting complex
NPQ: Non-photochemical quenching
ROS: Reactive oxygen species
SOD: Superoxide dismutase
