# Supplementary material for: Iron allocation to chloroplast proteins depends on the DNA-binding protein WHIRLY1
Source: Planta. 2025 Jun 17;262(2):32. doi: 10.1007/s00425-025-04736-8 (PMC12174181; doi:10.1007/s00425-025-04736-8)
Supplement: Supplementary file 1 — Supplementary file1 (PDF 496 KB) [file 425_2025_4736_MOESM1_ESM.pdf]

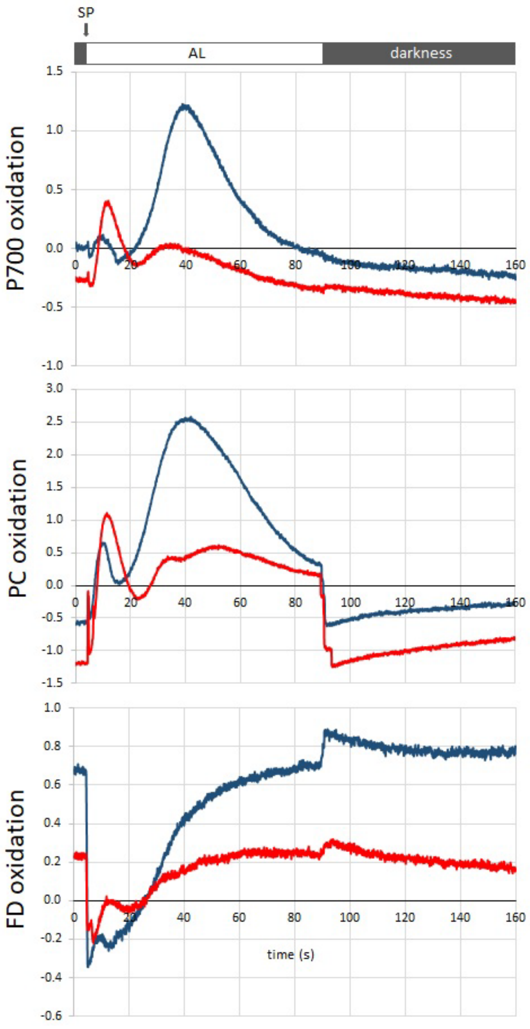

Fig. S1 Changes in the oxidation states of plastocyanin (PC), P700 and ferredoxin (Fd) in primary foliage leves of barley seedlings of the wild type (blue lines) and the W1-7 line (red lines) collected 10 days after sowing. Spectra were recoded by a Dual/KLAS-NIR spectrophotometer (Walz GmbH, Effeltrich, Germany).
Leaf segments were cut from dark-adapted seedlings (30 minutes darkness) immediately before measurements.
Pulses of saturating light (SP) were applied at 4 seconds after beginning of recording together with the onset of actinic illumination (AL) of 280 µmol m-2s-1. AL was switched off after 90 seconds. Each line represents an averaged run of four repititions.

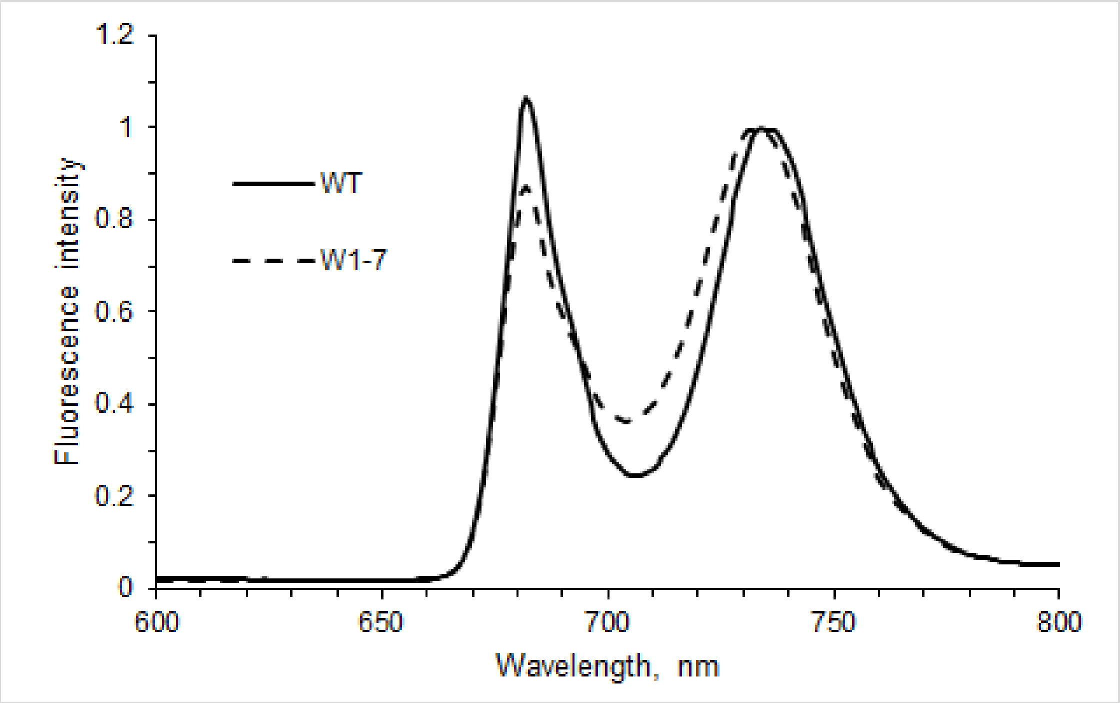

Fig. S2 77 K fluorescence emission spectra performed with thylakoids from wild-type (dotted line) and W1-1 plants (grey line). Samples contained 10 mg of chlorophyll. Emission was excited with light of 435 nm and was recorded from 650 to 800 nm using a Hitachi F-4500 FL fluorescence spectrophotometer (Higashiueno, Tokyo, Japan). For the isolation of thylakoids, leaves were ground with isotonic HEPES buffer (20 mM HEPES/NaOH, 5 mM $MgCl_2$, 10 mM $NaCO_3$, pH 7.0) supplemented with 330 mM sorbitol using a chilled mortar. The suspension was centrifuged for 5 min at 400 x g to remove cell debris. Subsequently, the supernatant was centrifuged for five minutes at 2.500 x g to sediment chloroplasts. Chloroplasts were osmotically broken to release thylakoids, which were diluted with HEPES buffer to a chlorophyll concentration of 10 µg/ml and frozen in liquid nitrogen inside a unique glass cuvette

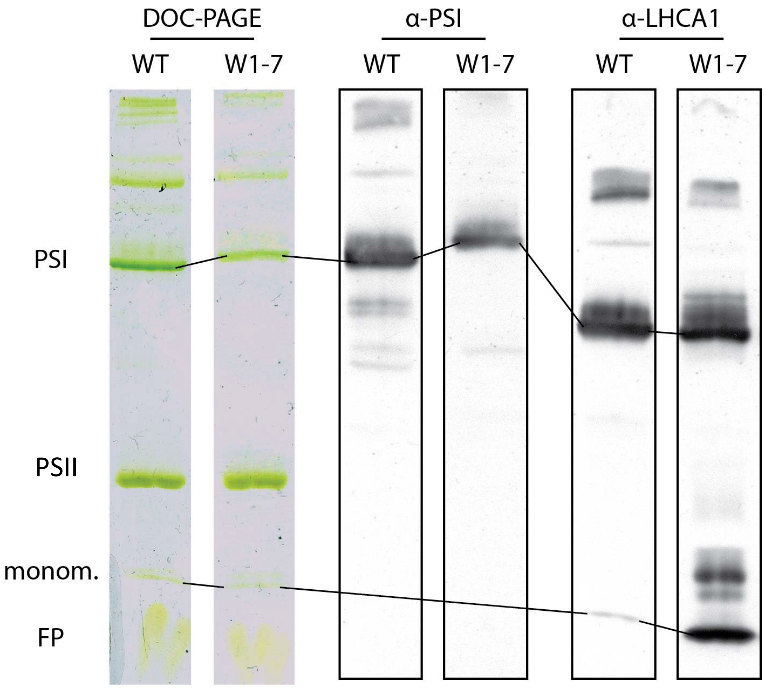

Fig. S3 Separation of thylakoid membrane complexes by DOC-PAGE. Clear native separation of complexes was performed after the solubilization of thylakoid membranes with 1% ß-dodecyl maltoside according to Järvi et al. (2011). PSI megacomplexes and monomers are indicated. The native gels were blotted and immunologically analysed with specific antibodies prepared against isolated PSI or LHCA1.

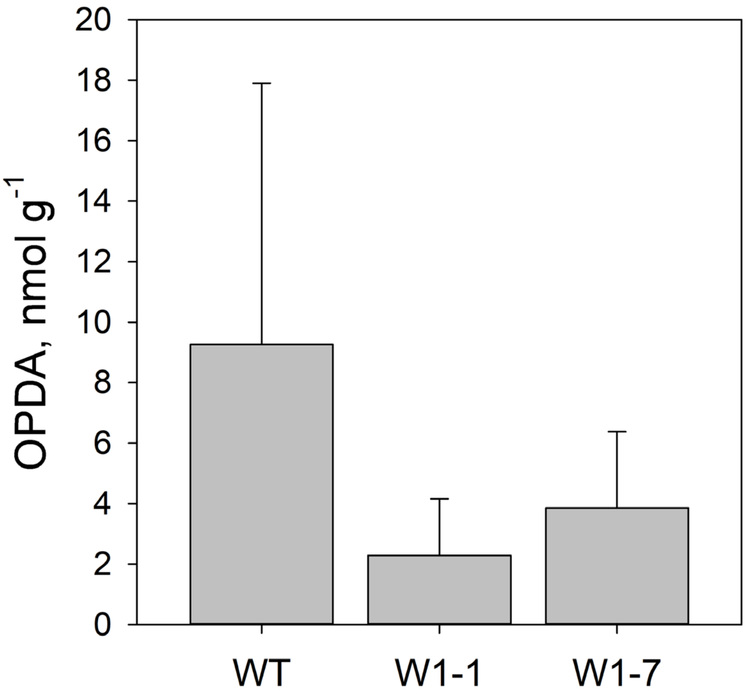

Fig. S4 The content of oxophytodienoic acid (OPDA) in pmol/g fresh weight of primary foliage leaves
of the barley wild type (WT) and the *HvWHIRLY* knockdown lines W1-1 and W-7. Quantification of OPDA was done using about 50 mg of leaf material that was extracted with 500 µl methanol supplied with 50 ng of ($^2H_6$)-OPDA as internal standard. After centrifugation, the supernatant was diluted with 9 volumes of water and subjected to solid phase extraction on HR-XC (Chromabond, Macherey-Nagel) column. After elution of OPDA with 900 µl acetonitrile, 10 µl of the eluate were subjected to ultraperformance liquid chromatography–tandem mass spectrometry (LC-MS/MS) according to Balcke et al. (2012). The content of OPDA was calculated using the ratio of analyte and internal standard peak heights.

G. Balcke, V. Handrick, N. Bergau, M. Fichtner, A. Henning, H. Stellmach, A. Tissier, B. Hause, A. Frolov (2012) An UPLC-MS/MS method for highly sensitive high-throughput analysis of phytohormones in plant tissues. Plant Methods 8: 47, doi:10.1186/1746-4811-8-47
