# Supplementary material for: Iron allocation to chloroplast proteins depends on the DNA-binding protein WHIRLY1
Source: Planta. 2025 Jun 17;262(2):32. doi: 10.1007/s00425-025-04736-8 (PMC12174181; doi:10.1007/s00425-025-04736-8)
Supplement: Supplementary file 2 — Supplementary file2 (PDF 472 KB) [file 425_2025_4736_MOESM2_ESM.pdf]

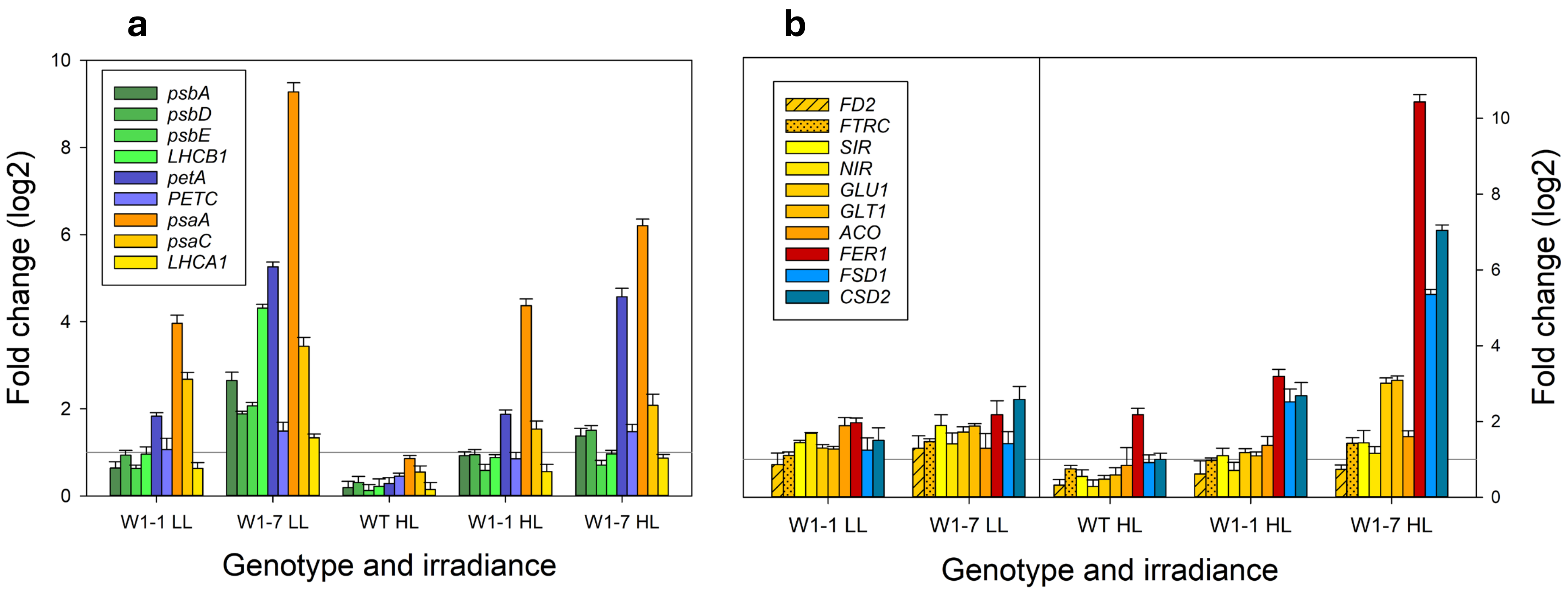

Fig. S5 Gene expression in primary foliage leaves of wild-type (WT) and W1 seedlings grown in continuous light of low (LL: 100 µmol m^{-2} s^{-1}) or high irradiance (HL: 350 µmol m^{-2} s^{-1}). **a** Expression of genes encoding the following photosynthesis-associated proteins: photosystem II center proteins (*psbA, psbD*) cytochrome f (*petA*), the Rieske protein of the cytochrome b_6f complex (PETC), subunits of photosystem I (*psaA, psaC, PSAF*), and subunits of the light-harvesting complexes (*LHCB1* and *LHCA1*). **b** Expression of genes encoding chloroplast iron-sulfur cluster proteins (yellow and orange) are *FD2, FTRC, SIR*, *NIR, GLU1, GLT1, ACO1*. *FSD1* and *CSD2* (blue) encode chloroplast superoxide dismutases. Expression levels were calculated relative to the expression levels in the wild type at low irradiance (LL) (set to 1 and represented by a horizontal line). Primers are listed in the Supplemental Table 1. ACO1, aconitase 1; CSD2, Cu/Zn superoxide dismutase; FD2, ferredoxin; FSD1, Fe superoxide dismutase; FER1, ferritin; FTRC, ferredoxin thioredoxin reductase; reductase; GLT1, glutamate synthase; GLU1, glutamine synthase; NIR, nitrite reductase; SIR, sulfite synthase
