# Supplementary material for: Iron allocation to chloroplast proteins depends on the DNA-binding protein WHIRLY1
Source: Planta. 2025 Jun 17;262(2):32. doi: 10.1007/s00425-025-04736-8 (PMC12174181; doi:10.1007/s00425-025-04736-8)
Supplement: Supplementary file 3 — Supplementary file3 (PPTX 120 KB) [file 425_2025_4736_MOESM3_ESM.pptx]

## Slide 1
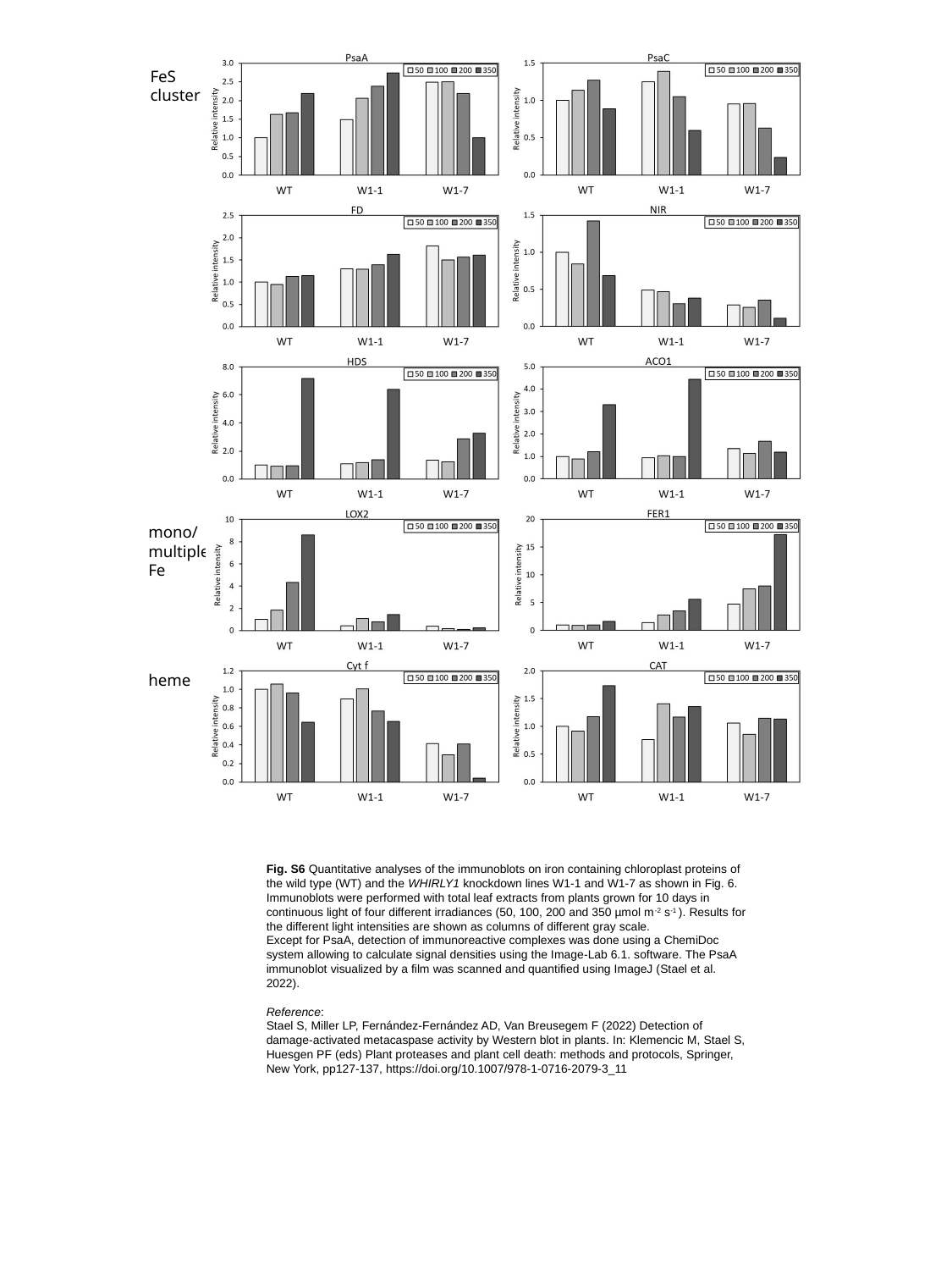

FeS
cluster
mono/
multiple
Fe
heme
Fig. S6 Quantitative analyses of the immunoblots on iron containing chloroplast proteins of the wild type (WT) and the WHIRLY1 knockdown lines W1-1 and W1-7 as shown in Fig. 6. Immunoblots were performed with total leaf extracts from plants grown for 10 days in continuous light of four different irradiances (50, 100, 200 and 350 µmol m-2 s-1 ). Results for the different light intensities are shown as columns of different gray scale.
Except for PsaA, detection of immunoreactive complexes was done using a ChemiDoc system allowing to calculate signal densities using the Image-Lab 6.1. software. The PsaA immunoblot visualized by a film was scanned and quantified using ImageJ (Stael et al. 2022).
Reference:
Stael S, Miller LP, Fernández-Fernández AD, Van Breusegem F (2022) Detection of damage-activated metacaspase activity by Western blot in plants. In: Klemencic M, Stael S, Huesgen PF (eds) Plant proteases and plant cell death: methods and protocols, Springer, New York, pp127-137, https://doi.org/10.1007/978-1-0716-2079-3_11
