# Supplementary material for: Iron allocation to chloroplast proteins depends on the DNA-binding protein WHIRLY1
Source: Planta. 2025 Jun 17;262(2):32. doi: 10.1007/s00425-025-04736-8 (PMC12174181; doi:10.1007/s00425-025-04736-8)
Supplement: Supplementary file 4 — Supplementary file4 (DOCX 21 KB) [file 425_2025_4736_MOESM4_ESM.docx]

**Supplemental Table S1** Genes and primers. PSI, photosystem I; PSII, photosystem II; LHC, light harvesting complex

| **gene** | **protein** | ***Arabidopsis thaliana* accession number** | **barley gene number**  **Acc. No** | **primer 1** | **primer 2** |
| --- | --- | --- | --- | --- | --- |
| *ACO3* | ACONITASE | At2g05710 (ACO3) | AK354442 | CTCCGAAGTTGGCTGAGATAC | GCATAGCTGCAAGATCAACAA |
| *CSD2* | CuZn-SUPEROXIDE DISMUTASE 2 | At2G28190 | HM537232 | gcagttgttgggagagcatt | cttccaccagcatttccagt |
| *FD2* | FERREDOXIN 2 | At1G60950 | XP_044956456 | gcttaccctgcctctctttg | atgcatcgagcaattcattc |
| *FSD3* | Fe-SUPEROXIDE DISMUTASE | At5G23310 | AK375983 | AAGGATTTCGGCTCCTTTGT | GTATGAACCACCGCGAGTTT |
| *FER1* | FERRITIN 1 | At5G01600 | EF440353 | ccctcaacgagcagatcaat | gcaaatcccttgagagcaa |
| *FRO7* | ferrric chelate reductase | At5g49740 | AK370505.1 | AAGTCGGAATAGCAAACTTACCC | ACTACGTAGAGCTGGTGGGTGTA |
| *FTRC* | FERREDOXIN-THIORE­DOXIN REDUCTADE | At2G04700 | XM_045097840 | GAAAGGAAGGAGTGCCATTG | CCTTGATCTCCTCCAAGCTG |
| *GLU1* | FD-DEPENDANT GLUTAMATE SYNTHASE 1 | At5G04140 | XM_045108608 | TGGGCTAGATGTCCTTTTGG | CAACTGGGCAATTGTTTGTG |
| *GLT1* | GLUTAMATE SYNTHASE 1 | At5G53460 | XM_045120740 | CCCTGGAATGATGTTACTCG | TGTCTCTTGAGCCATTCTCC |
| *IRO2* | bHLH transcription factor 056 | Rice: Os01g72370 | AB206536.1 XM_045122540.1 | CTCGTCCACTTCTGCTTTCC | AAAATGCGGGACACTCTTTG |
| *LHCA1* | subunit A of LHCI | **AT3G54890** | AF218305 | ccggagaagaagaagtaccc | cgttcttgatctccttgagc |
| *NEET* | iron-sulfur transfer protein | At5g51720 | AK358658.1  XM_045094294.1 | GTGATGGAAGCCATGTGAAG | ccactcaacacaaattgagga |
| *NIR* | NITRITE REDUKTASE | At2g15620 | AK371794 | ctggtctccttctcctgatga | cggcattgcctactttacatc |
| *petA* | cytochrome f | At2G15620 | NC_008590.1 | tatacctcccggaccagaac | acctcccacattaggattgc |
| *PETC* | chloroplast Rieske protein | At4g03280 | AK361408 | TCGAGGATTGGCTCAAGAC | TCCACCACAAGGTAGGTAGGAT |
| *PIC1* | iron permease in the plastid envelope, TIC21 | At2g15290 | AK366427  XM_045096830.1 | TACCGAGCAGACAATTGAGC | AAACAAACGTGCACACCAAC |
| *psaA* | subunit A of PSI | AtCg00350 | NC_008590.1 | cttttcctaatcgcaggtca | aagacccttatggccttgtc |
| *psaC* | subunit C of PSI | AtCg01060 | NC_008590.1 | CGAGAACCGAAGATTGTGTG | CATGCTACGGGTTGTTTCAG |
| *PSAF* | subunit F of PSI | At1g31330 | U08135 | CGTGCTCTTCCTCTACATCG | GTCGATGATGATCTCCCTCA |
| *psbA* | subunit A of PSII | AtCg00020 | NC_008590.1 | ctgcttggcctgtagtagga | cgcgaccttgactatcaact |
| *psbD* | subunit D of PSII | AtCg00270 | NC_008590.1 | atttgaacttgctcggtctg | ttgccccagtggataaataa |
| *PSBS* | subunit S of PSII | At1g44575 | AK359183 | CTGTTCGGGTTCACCAAGT | GATGATCTCCCCGATGATG |
| *SIR* | SULFITE REDUKTASE | At5g04590 | AK372042 | TCAAGCTGGTCTGTTGGAAC | TGATGCCAACCTTGTCGAAA |
|  |  |  |  |  |  |
| *GAPDH**  *housekeeping* | glyceraldehyde-3-phophate dehydrogenase | At1g13440 | AK355370 | TTCGGCGAGAAGCCAGTTA | CCTCACCCCACGGGATCT |

Barley plastome sequence: NC_008590.1

*Acta Physiol Plant (2012) 34:1723-1733)

|  |  |  |
| --- | --- | --- |
