# Supplementary material for: Iron allocation to chloroplast proteins depends on the DNA-binding protein WHIRLY1
Source: Planta. 2025 Jun 17;262(2):32. doi: 10.1007/s00425-025-04736-8 (PMC12174181; doi:10.1007/s00425-025-04736-8)
Supplement: Supplementary file 5 — Supplementary file5 (DOCX 14 KB) [file 425_2025_4736_MOESM5_ESM.docx]

**Supplementary Table S2**. Results of a two way ANOVA for Ф(NPQ) and Ф(NO) using Sigmaplot 13 (Systat Software GmbH, Erkrath, Germany). Since (II) + Ф(NPQ) + Ф(NO) = 1, analysis of the data for Ф(II) was omitted.

**Variable Ф(NPQ)**

| **Source of variation** | **DF** | **F** | **P** |
| --- | --- | --- | --- |
| Genotype | 2 | 226.369 | <0.001 |
| Irradiance | 3 | 2.093 | 0.128 |
| Genotype x irradiance | 6 | 4.152 | 0.005 |

**Variable Ф(NO)**

| **Source of variation** | **DF** | **F** | **P** |
| --- | --- | --- | --- |
| Genotype | 2 | 1.427 | 0.260 |
| Irradiance | 3 | 12.215 | <0.001 |
| Genotype x irradiance | 6 | 2.660 | 0.040 |
